# Biodegradable Zn‐5Dy Alloy with Enhanced Osteo/Angio‐Genic Activity and Osteointegration Effect via Regulation of SIRT4‐Dependent Mitochondrial Function

**DOI:** 10.1002/advs.202307812

**Published:** 2024-01-19

**Authors:** Yue Han, Xian Tong, Runqi Zhou, Yilin Wang, Yuge Chen, Liang Chen, Xinhua Hong, Linmei Wu, Zhiqiang Lin, Yichi Zhang, Xuejia Zhang, Chaoming Hu, Bin Li, Yifan Ping, Zelin Cao, Zhou Ye, Zhongchen Song, Yuncang Li, Cuie Wen, Yongsheng Zhou, Jixing Lin, Shengbin Huang

**Affiliations:** ^1^ Institute of Stomatology School and Hospital of Stomatology Wenzhou Medical University Wenzhou 325027 China; ^2^ Department of Dentistry Faculty of Medicine and Dentistry University of Alberta Edmonton T6G2R3 Canada; ^3^ Applied Oral Sciences and Community Dental Care Faculty of Dentistry University of Hong Kong Hong Kong 999077 China; ^4^ Department of Periodontology Ninth People's Hospital Shanghai Jiao Tong University School of Medicine Shanghai 200125 China; ^5^ School of Engineering RMIT University Melbourne VIC 3001 Australia; ^6^ Department of Prosthodontics National Center for Stomatology National Engineering Research Center of Oral Biomaterials and Digital Medical Devices National Clinical Research Center for Oral Disease Beijing Key Laboratory of Digital Stomatology Research Center of Engineering and Technology for Computerized Dentistry Ministry of Health Peking University School and Hospital of Stomatology Beijing 100081 China

**Keywords:** biodegradable zinc alloy, bone fracture, osteointegration, rare earth dysprosium (Dy), SIRT4

## Abstract

Zinc (Zn)–dysprosium (Dy) binary alloys are promising biodegradable bone fracture fixation implants owing to their attractive biodegradability and mechanical properties. However, their clinical application is a challenge for bone fracture healing, due to the lack of Zn–Dy alloys with tailored proper bio‐mechanical and osteointegration properties for bone regeneration. A Zn‐5Dy alloy with high strength and ductility and a degradation rate aligned with the bone remodeling cycle is developed. Here, mechanical stability is further confirmed, proving that Zn‐5Dy alloy can resist aging in the degradation process, thus meeting the mechanical requirements of fracture fixation. In vitro cellular experiments reveal that the Zn‐5Dy alloy enhances osteogenesis and angiogenesis by elevating SIRT4‐mediated mitochondrial function. In vivo Micro‐CT, SEM‐EDS, and immunohistochemistry analyses further indicate good biosafety, suitable biodegradation rate, and great osteointegration of Zn‐5Dy alloy during bone healing, which also depends on the upregulation of SIRT4‐mediated mitochondrial events. Overall, the study is the first to report a Zn‐5Dy alloy that exerts remarkable osteointegration properties and has a strong potential to promote bone healing. Furthermore, the results highlight the importance of mitochondrial modulation and shall guide the future development of mitochondria‐targeting materials in enhancing bone fracture healing.

## Graphical abstract

Zinc (Zn)–dysprosium (Dy) binary alloys are promising biodegradable bone fracture fixation implants. The Zn‐5Dy alloy exerts remarkable osteogenesis and angiogenesis properties dependent on the upregulation of SIRT4‐mediated mitochondrial events. This study highlights the importance of mitochondrial modulation and guides the future development of mitochondria‐targeting biodegradable metal materials in enhancing bone fracture healing.

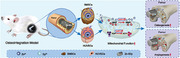

## 1 Introduction

In recent years, the number of patients with bone fractures caused by injuries, tumors, infections, traffic accidents, and other external causes has increased, becoming an increasingly severe concern impacting human health.^[^ ^1^, ^2^ ^]^ Conventional orthopedic implants made of permanent rigid metals such as stainless steel and titanium (Ti) alloys are susceptible to the stress‐shielding effect, shielding the mechanical stress that is ordinarily loaded onto the surrounding bone, thus leading to bone atrophy and implant loosening.^[^ ^3^ ^]^ Additionally, these types of implants require secondary surgical removal once they have served their purpose due to their non‐degradability.^[^ ^4^ ^]^ In contrast, biodegradable metal materials are widely studied as candidates for innovative bone‐fracture fixation systems because of their biodegradability and potential biofunctionality. In particular, zinc (Zn) and some of its alloys have received increasing attention in recent years due to their moderate degradation rate, mechanical strength comparable to or greater than that of cortical bone, biocompatibility, and biofunctionalities. Zn is an essential trace element in the human body that is required for normal physiological functions and biochemical metabolism.^[^ ^5^ ^]^ In addition, Zn stimulates bone formation and mineralization, and 85% of Zn is located in the bone and muscle tissues of the body.^[^ ^6^ ^]^ Furthermore, Zn deficiency can slow bone metabolism and development.^[^ ^7^ ^]^ As a result, Zn alloys can be considered viable biodegradable orthopedic implants for bone‐fracture fixation. However, cast pure Zn and its alloys have low mechanical strength and degradation rates, making it challenging for them to meet the requirements of bone‐fracture fixation materials.^[^ ^8^ ^]^ A previous study^[^ ^9^ ^]^ indicated that adding the rare earth element dysprosium (Dy) improved the mechanical strength, accelerated the degradation rate, and enhanced the in vitro biocompatibility of pure Zn. Nevertheless, the fundamental mechanisms by which Dy improves biocompatibility and whether it facilitates fracture healing in vivo are unknown. Therefore, a fundamental investigation to uncover the mechanisms is crucial.

A bone‐fixation system is used to fix fractured bones and enhance healing in the load‐bearing skeletal region. Bone‐fracture healing involves two main processes: osteogenesis and angiogenesis.^[^ ^10^ ^]^ Osteoblasts play an essential role in the maintenance and integration of bone, interacting with osteoclasts to maintain bone homeostasis.^[^ ^11^ ^]^ Angiogenesis refers to the formation of new blood vessels through the sprouting, proliferation, and migration of vascular endothelial cells based on existing blood vessels.^[^ ^12^, ^13^ ^]^ In addition, angiogenesis and osteogenesis synergize during bone formation and reconstruction in bone‐fracture healing.^[^ ^14^, ^15^, ^16^, ^17^, ^18^ ^]^ It is worth noting that mitochondria play a central role in osteoclast differentiation^[^ ^19^ ^]^ and endothelial cell migration^[^ ^20^, ^21^ ^]^ as the energy factories of cells. During osteogenic differentiation, mitochondrial membrane potential, respiratory enzyme complexes, oxygen consumption, and intracellular adenosine triphosphate (ATP) content are all significantly elevated.^[^ ^22^ ^]^ Additionally, mitochondrial metabolism influences endothelial cell activity and angiogenesis is dependent on the mitochondrial‐shaping protein optic atrophy 1.^[^ ^23^ ^]^ Therefore, exploring the involvement of mitochondria in osteointegration and angiogenesis would aid in elucidating the molecular mechanism by which Zn‐5Dy promotes fracture healing.

As a protein family with nicotinamide adenine dinucleotide (NAD)+‐dependent deacetylase or adenosine diphosphate (ADP)‐ribosyltransferase activity, sirtuins (SIRTs) generate various post‐translational protein modifications, governing essential physiological processes such as cell cycle, autophagy, and gene expression.^[^ ^24^, ^25^ ^]^ Among mammals, SIRT3, SIRT4, and SIRT5 are predominantly localized inside mitochondria, regulating energy metabolism and stress response, and participating in the balance between oxidation and antioxidants.^[^ ^26^ ^]^ The SIRT3 knockdown can cause decreases in mitochondrial density, membrane potential, and alkaline phosphatase (ALP) activity in osteoblasts;^[^ ^27^ ^]^ SIRT4 can regulate the differentiation of rat papillary cells by promoting mitochondrial function.^[^ ^28^ ^]^ Tao et al.^[^ ^29^ ^]^ also suggested that SIRT4 inhibited the phosphatidylinositol‐3‐kinase/Akt (a serine/threonine protein kinase, also called protein kinase B or PKB)/nuclear factor (NF)‐κB signaling pathway and mitigated damage to oxidized low‐density lipoprotein‐induced human umbilical vein endothelial cells (HUVECs); downregulation of SIRT4 can cause mitochondrial decoupling, leading to endothelial dysfunction and ADP/ATP translocase 2(adenine nucleotide translocator‐2) inhibition.^[^ ^30^ ^]^ Although SIRTs have been shown to play a critical role in regulating various vital physiological processes, little is known about the role that they probably play in regulating endothelial cells and osteoblast mitochondrial function during osteointegration and angiogenesis by the Zn‐5Dy alloy.

In this study, biodegradable Zn‐xDy (*x* = 1, 3, and 5 wt.% hereafter) alloy plates were prepared via casting and hot‐rolling for bone‐fixation system applications. The mechanical properties, degradation behavior, cytotoxicity, angiogenesis, SIRT‐regulated mitochondrial function, and osteointegration of the Zn–Dy alloys were systematically evaluated in vitro and in vivo, and compared with those of pure Zn. In particular, the following four issues have been explored: 1) mechanical properties and degradation behavior of the Zn‐5Dy alloy as bone‐fracture fixation materials; 2) SIRT‐regulated mitochondrial mechanisms during osteoblast differentiation promoted by the Zn‐5Dy alloy; 3) SIRT‐regulated mitochondrial mechanisms during pathogenesis promoted by the Zn‐5Dy alloy; and 4) the Zn‐5Dy alloy promotion of angiogenesis and osteointegration through a SIRT‐dependent mitochondrial mechanism in an animal model.

## 2 Results

### 2.1 Mechanical Properties and Degradation Behavior of Zn‐5Dy

**Figure 1:**
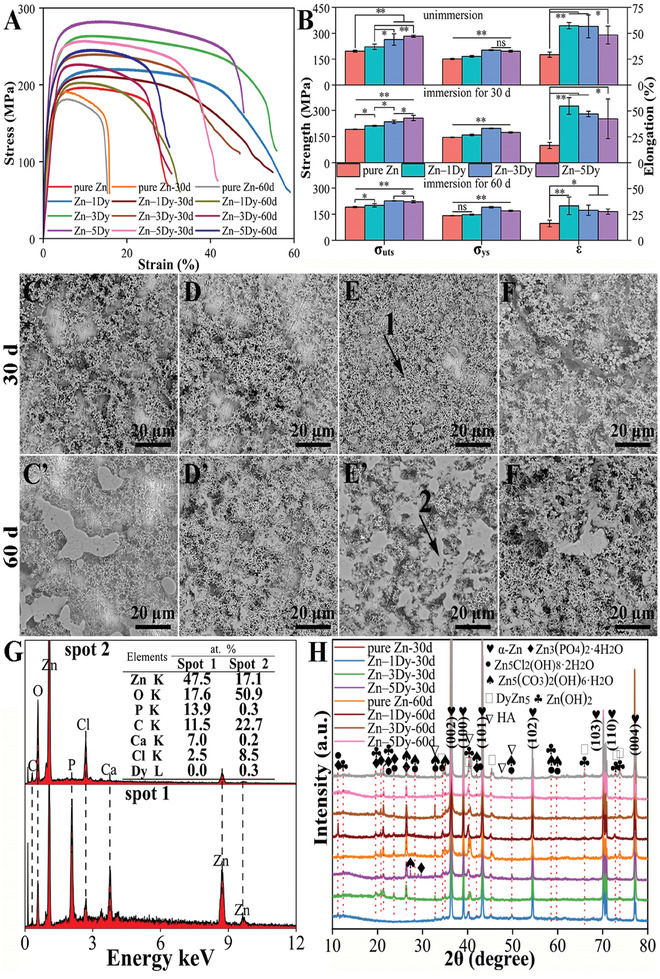
Tensile properties of HR Zn‐xDy alloys, SEM images, EDS spectra, and XRD patterns of corrosion products on HR samples after 30 and 60 d of immersion in Hanks’ solution: A) tensile stress–strain curves; B) bar charts of σ_uts_, σ_ys_, and ε; C–F’) SEM images of corrosion products on Zn‐xDy alloy surfaces; G) EDS spectra of corrosion products on Zn‐3Dy alloy surfaces after immersion for 30 d (Spot 1) and 60 d (Spot 2); and H) XRD patterns of corrosion products on sample surfaces. ^*^p < 0.05, ^**^p < 0.01.

**Figure** **1**A shows tensile stress–strain curves for hot‐rolled (HR) Zn‐xDy alloys after 0, 30, and 60 days of immersion in Hanks’ solution, while Figure 1B depicts the bar charts of the corresponding tensile properties. Prior to immersion testing, the ultimate tensile strength (σ_uts_) of the HR samples exhibited a gradual increase with the addition of Dy content. In contrast, both the yield strength (σ_ys_) and elongation (ε) demonstrated an initial increase followed by a decrease. The HR Zn‐3Dy alloy displayed the most favorable mechanical properties, while the HR Zn‐5Dy alloy exhibited marginally lower properties, with a σ_uts_ of 283.0 ± 6.6 MPa, a σ_ys_ of 196.3 ± 5.4 MPa, and an ε of 48.3 ± 8.6%. Following 30 d of immersion, the mechanical properties of the HR samples showed a decreasing trend with increasing Dy content at various levels. Pure Zn experienced the most significant decrease, with a 43.2% reduction in ε. The HR Zn‐5Dy alloy showed a σ_uts_ of 256.0 ± 15.4 MPa, a σ_ys_ of 172.9 ± 3.6 MPa, and an ε of 42.3 ± 19.1% after 30 d immersion, decreases of 9.5%, 11.9%, and 12.4%, respectively, compared with the tensile samples that did not undergo immersion. The mechanical properties of the HR samples were further reduced by extending the immersion time to 60 d. The Zn‐5Dy alloy maintained a σ_uts_ of 221.0 ± 6.5 MPa, a σ_ys_ of 169.1 ± 3.6 MPa, and an ε of 27.7 ± 2.5%, indicating the high mechanical stability of the HR Zn‐5Dy alloy in Hanks’ solution.

Figure 1C–F’ displays scanning electron microscopy (SEM) images of corrosion products on the HR Zn‐xDy (*x* = 0, 1, 3, and 5) alloy surfaces after 30 and 60 d of immersion in Hanks’ solution. With increasing Dy content, the amount of corrosion products on the HR sample surfaces showed a gradually increasing trend. With the extension of immersion time from 30 d to 60 d, the amount of corrosion products on the sample surfaces also increased. In addition, the morphology of the corrosion products gradually changed from granular and flocculent to granular and flaky. Figure 1G presents the energy‐dispersive X‐ray spectroscopy (EDS) profiles of granular (Spot 1) and flaky (Spot 2) corrosion products. The granular corrosion products consist of high levels of phosphorus (P) and calcium (Ca), with a small amount of chlorine (Cl), whereas the flaky corrosion products comprise large amounts of carbon (C) and Cl, and trace amounts of P, Ca, and Dy. Figure 1H shows X‐ray diffraction (XRD) patterns of the corrosion products on the sample surfaces after 30 and 60 d of immersion. The corrosion products primarily consist of α‐Zn, Zn_3_(PO_4_)_2_·4H_2_O, Zn_5_Cl_2_(OH)_8_·2H_2_O, Zn_5_(CO_3_)_2_(OH)_6_·H_2_O, Zn(OH)_2_, Ca_10_(PO_4_)_6_(OH)_2_ (HA), and a small quantity of DyZn_5_ phases. With the extension of immersion time from 30 d to 60 d, the diffraction peak intensities of the corrosion products on the sample surfaces showed increasing trends except for the α‐Zn and DyZn_5_ phases, indicating that the number of corrosion products on the surfaces tended to increase, which is consistent with their SEM morphologies. Based on EDS and XRD results, it can be inferred that Spot 1 may be Zn_3_(PO_4_)_2_·4H_2_O and HA phases, while Spot 2 may comprise Zn_5_(CO_3_)_2_(OH)_6_·H_2_O and Zn_5_Cl_2_(OH)_8_·2H_2_O phases.

### 2.2 Cytocompatibility of Zn‐5Dy

Figure S1 (Supporting Information) shows the concentrations of Zn^2+^ and Dy^2+^ ions in Zn‐xDy (*x* = 0, 1, 3, and 5) extracts from different media: α‐MEM, DMEM/F‐12, DMEM/sodium pyruvate[−], and DMEM/sodium pyruvate[+]. The concentrations of Zn^2+^ and Dy^2+^ ions increased with an increase in Dy content.

**Figure 2:**
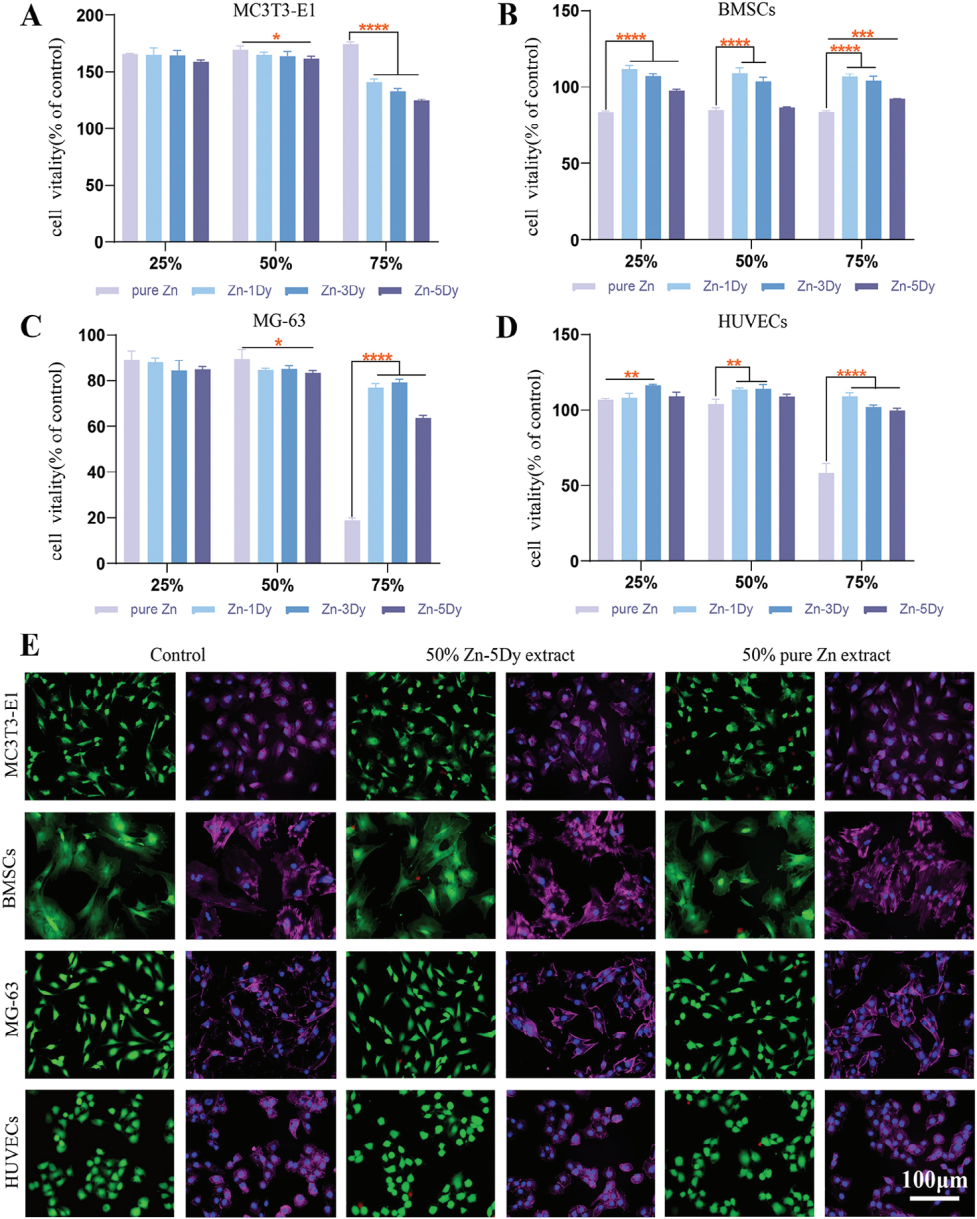
A–D) The cell viability of Zn‐xDy (*x* = 0, 1, 3, and 5) alloy extracts at 25%, 50%, and 75% concentrations after culture with four cell lines s for 3 d: A) MC3T3‐E1; B) BMSCs; C) MG‐63; and D) HUVECs; E) cytoskeletal staining of MC3T3‐E1, BMSCs, MG‐63, and HUVECs after culturing with 50% concentration extracts of Zn‐5Dy, pure Zn, and control. ^*^p < 0.05, ^**^p < 0.01, ^***^p < 0.001, ^****^p < 0.0001.

**Figure** **2**A–D presents the cell viabilities of a mouse embryo osteoblast precursor cell lines (MC3T3‐E1), bone marrow mesenchymal stem cells (BMSCs), human osteosarcoma cells (MG‐63), and HUVECs cells after being co‐cultured with HR Zn‐xDy (*x* = 0, 1, 3, and 5) alloy extracts at 25%, 50%, and 75% concentrations for 72 h. For MC3T3‐E1 cells, cell viability exceeded 100% in all extract groups. No statistical difference was found between groups at a 25% concentration. At a 50% concentration, cell viability for Zn‐xDy groups was lower than that of the pure Zn group, while at a 75% concentration, Zn‐xDy groups showed a more significant decrease in cell viability with a decreasing trend with increasing Dy content. For BMSCs, the cell viability for all Zn‐xDy alloy concentration extracts was higher than the pure Zn group and also showed a decreasing trend with increasing Dy content. For MG‐63 cells, all groups had cell viability between 60% and 100%, except for the 75% extract from the pure Zn group. No statistical differences were found between groups at 25% concentration. The cell viability of the Zn–1Dy alloy groups was lower than that of the pure Zn group at 50% concentration, while it was higher than that of the pure Zn group and showed a decreasing trend at a concentration of 75%. For HUVECs, the cell viability of all groups was higher than 100% except for the 75% concentration extract of the pure Zn group, and the cell viability of different concentrations of Zn–1Dy groups showed a trend of first increasing and then decreasing with an increase in Dy content. The cell viability results suggest that Zn‐xDy alloy extracts have good cytocompatibility and a pro‐proliferative effect. Figure 2E shows cytoskeletal staining of MC3T3‐E1, BMSCs, MG‐63, and HUVECs after culturing with 50% concentration extracts of Zn‐5Dy, pure Zn, and control. It can be seen that the four types of cells cultured with the 50% concentration extracts of Zn‐5Dy, pure Zn, and control show similar shapes and spreading, suggesting that the 50% concentration extracts of HR Zn‐5Dy and pure Zn are biologically safe in relation to these four types of cells.

For the cell viability of Zn‐xDy alloy extracts at 25%, 50%, and 75% concentrations after culture with four cell lines for 1 and 7 d, there was no significant difference between the 25% and 50% concentration extracts on the three bone‐related cells, except for a significant decrease in cell viability at the 75% concentration extract (Figure S2, Supporting Information). It is worth mentioning that the cell viability of HUVECs in the 50% concentration extract at 1 and 7 d significantly decreased compared to that in the 25% concentration extract. Thus, the 25% concentration extract with the best cell viability toward four cell lines was selected for further cell testing. Meanwhile, the Zn‐5Dy alloy extract at 25% concentration exhibited the highest expression of ALP activity among the Zn‐xDy alloys and pure Zn (Figure S3, Supporting Information), suggesting the best regulating osteogenic differentiation ability. Therefore, the 25% concentration of Zn‐5Dy extract was employed for subsequent cell experiments.

### 2.3 Influence of Zn‐5Dy on Osteogenic Differentiation of MC3T3‐E1 Cells

**Figure 3:**
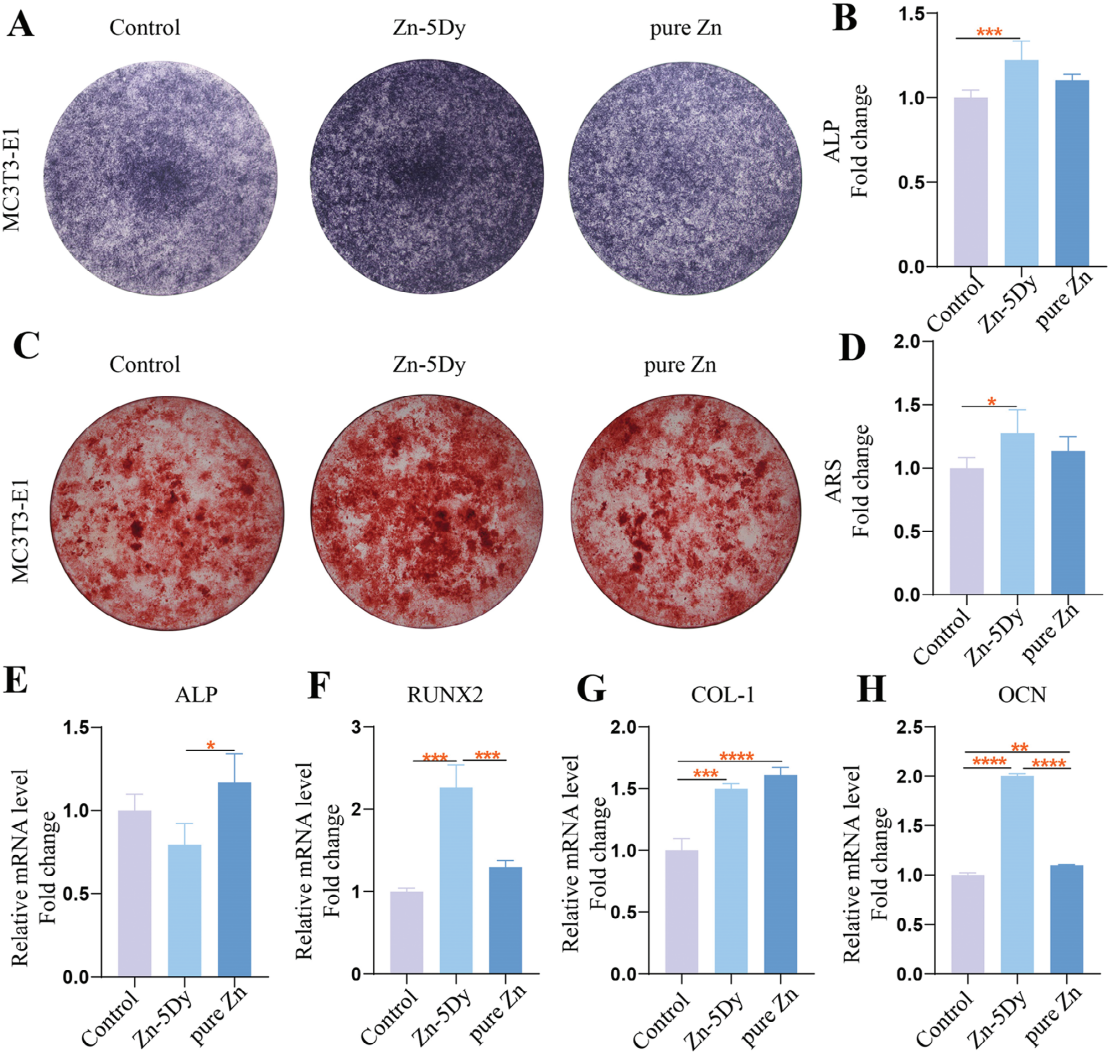
Osteogenic differentiation of 25% concentration extracts of HR Zn‐5Dy, pure Zn, and control toward MC3T3‐E1 cells: A) ALP staining of MC3T3‐E1 cells; B) quantitative analysis of ALP staining of MC3T3‐E1 cells; C) ARS staining of MC3T3‐E1 cells; D) quantitative analysis of ARS staining of MC3T3‐E1 cells; and E–H) relative gene expression levels of ALP, RUNX2, COL‐1, and OCN of MC3T3‐E1 cells. ^*^p < 0.05, ^**^p < 0.01, ^***^p < 0.001, ^****^p < 0.0001.

**Figure** **3**A–D shows osteogenic differentiation of 25% concentration extracts of the HR Zn‐5Dy, pure Zn, and control toward MC3T3‐E1. As shown in Figure 3A, the control and pure Zn groups stained lightly and there are still large blank areas at the bottom of the pores, but the Zn‐5Dy group stained blue almost all over the bottom of the pores, showing clear positive staining that indicates very high expression of ALP after 7 d of mineralization induction. Figure 3B shows the amounts of ALP staining in each group. The Zn‐5Dy group demonstrated higher ALP expression than the control group and about the same as the pure Zn group. Furthermore, as demonstrated by alizarin red S (ARS) staining in Figure 3C, after 21 d of mineralization induction in MC3T3‐E1 cells, there were only a few nodules in the control group. The staining of the control group is lighter and there are more blank areas to the naked eye, while the number of mineralized nodules in the pure Zn group and Zn‐5Dy group increased significantly, and in the overall view, the staining is darker in the Zn‐5Dy group than the other two groups. Figure 3D shows the amount of Ca in each group. The Zn‐5Dy group has more Ca than the control group and is about the same as the pure Zn group, consistent with the quantitative results of ALP staining. Figure 3E–H shows the expression levels of four critical genes relative to osteogenesis: ALP, COL‐1, OCN, and RUNX2. Except for ALP, the expression levels of the other three genes in the Zn‐5Dy group are significantly higher than those in the control groups, revealing that the Zn‐5Dy groups upregulated these osteogenesis‐related genes more than the control group. In addition, the RUNX2 and OCN expression levels of the Zn‐5Dy group were significantly higher than those of the pure Zn group. These results show that Zn‐5Dy induced stronger differentiation of MC3T3‐E1 cells than the control and pure Zn groups.

### 2.4 Impact of Zn‐5Dy on SIRT4‐Dependent Mitochondrial Function of MC3T3‐E1 Cells

**Figure 4:**
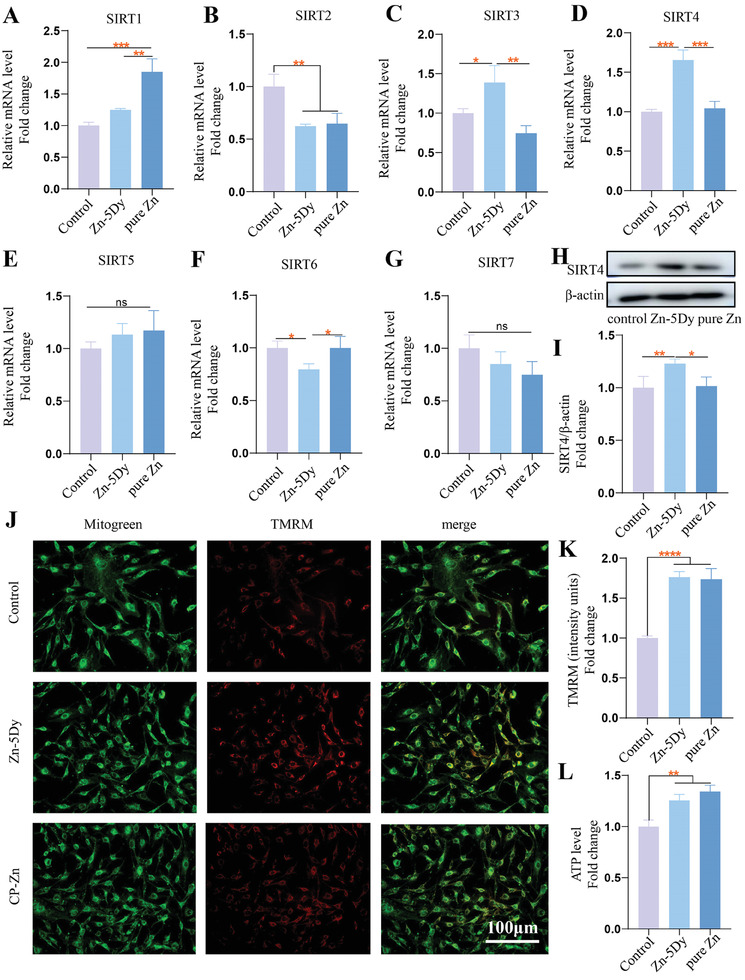
A–G) Expression of SIRT1–SIRT7 during osteogenic induction of MC3T3‐E1 cells in a mineralization‐inducing culture medium containing 25% concentration extracts of HR Zn‐5Dy, pure Zn, and control; H,I) western blotting; J) representative images of TMRM staining; K) quantitative analysis of TMRM fluorescence; and L) ATP level. ^*^p < 0.05, ^**^p < 0.01, ^***^p < 0.001, ^****^p < 0.0001.

**Figure** **4**A–G shows the expression of SIRTs during osteogenic induction of MC3T3‐E1 in a mineralization‐inducing culture medium containing 25% concentration extracts of the HR Zn‐5Dy, pure Zn, and control. Among the seven genes, the protein expression of SIRT3 and SIRT4 in the Zn‐5Dy group was higher than in the control and the pure Zn groups. Furthermore, the expression of SIRT4 was raised by a higher multiple than that of SIRT3. Figure 4H,I shows western blotting of HR Zn‐5Dy, pure Zn, and control. The Zn‐5Dy exhibits clearer upregulation of SIRT4 than that of the control, while the pure Zn group shows slight upregulation. Figure 4J,K shows the mitochondrial membrane potential (MMP) levels of MC3T3‐E1 cells during the osteogenic induction process. The tetramethylrhodamine methyl ester (TMRM) fluorescence increased significantly in the Zn‐5Dy group, indicating increased MMP. Figure 4L shows the ATP levels in osteoblasts‐treated extracts of Zn‐5Dy, pure Zn, and control. Similar to pure Zn, the Zn‐5Dy group shows a clearly enhanced ATP level compared to the control group. Overall, these results indicate an effective role of the Zn‐5Dy alloy in regulating the expression of SIRT4 and the mitochondrial function of MC3T3‐E1 cells.

### 2.5 Effect of Zn‐5Dy on MC3T3‐E1 Cell Osteogenesis via SIRT4

**Figure 5:**
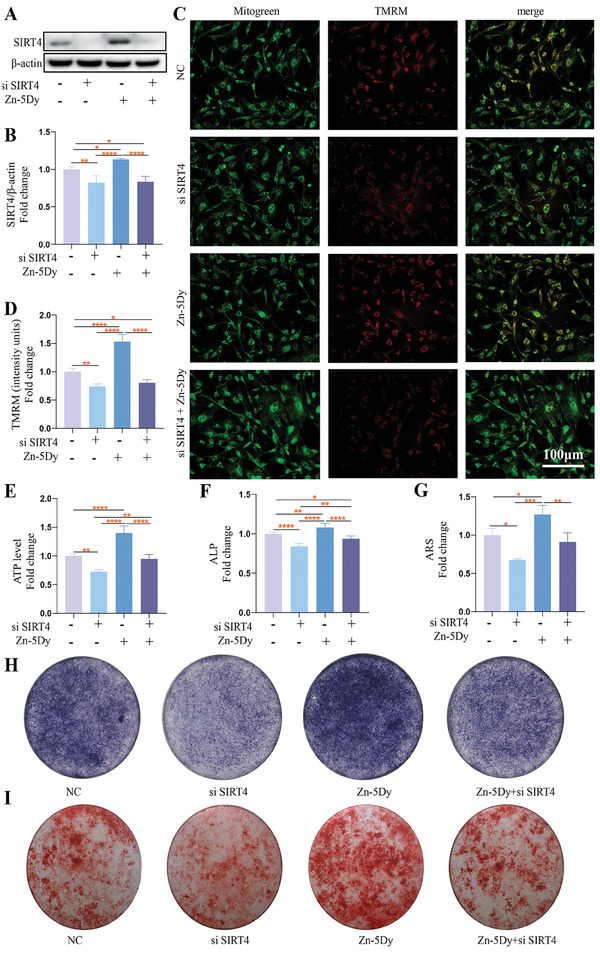
Osteogenic differentiation of MC3T3‐E1 cells after treating with osteogenic induction medium containing 25% concentration extract of Zn‐5Dy via regulating mitochondrial function through SIRT4: A) western blotting; B) quantitative analysis of western blotting; C) representative images of TMRM staining in assigned groups; D) quantitative analysis of TMRM; E) ATP levels in assigned groups; F) quantitative analysis of ALP staining; G) quantitative analysis of ARS staining; H) ALP staining; and I) ARS staining. ^*^p < 0.05, ^**^p < 0.01, ^***^p < 0.001, ^****^p < 0.0001.

**Figure** **5**A,B shows western blotting of osteoblasts treated with the HR Zn‐5Dy extracts and si SIRT4. It can be seen that the Zn‐5Dy extracts promoted the expression of SIRT4, but did not upregulate the expression of SIRT4 when osteoblasts were transfected with si SIRT4. Figure 5C–E indicates increases in MMP and ATP levels in osteoblasts mediated by the Zn‐5Dy extracts, which was inhibited by pre‐transfection of si SIRT4. Figure 5F–I shows si SIRT4 partially reversed ALP activity and the formation of mineralized nodules, but these were promoted by the Zn‐5Dy extract. Quantitative analysis also confirmed significant inhibition of mineralized nodule formation in the si SIRT4+Zn‐5Dy group. Collectively, these data suggest that Zn‐5Dy promotes osteoblast differentiation by upregulating SIRT4.

### 2.6 Effect of SIRT4 on Osteogenic Differentiation of MC3T3‐E1 Cells

Figure S4A–D (Supporting Information) shows ALP staining and the corresponding quantitative analysis results. The expression of ALP in MC3T3‐E1 cells induced by a mineralization‐inducing culture medium that contained 10 mm β‐glycerophosphate (G5704, Sigma, Germany) and 50 mm ascorbic acid (A4544, Sigma, Germany) (hereafter denoted VG) is higher than that of cells cultured in ordinary medium. The si SIRT4 group shows a significant decrease in ALP expression compared with the control group and mineralization induction significantly reversed these results caused by si SIRT4 transfection. Figure S4E–H (Supporting Information) shows ARS staining and the corresponding quantitative analysis. The VG group has a large number of mineralized nodules, while the control group has almost no mineralized nodules. In the absence of mineralization induction, there are almost no mineralization nodules in the control group and si SIRT4 group, and no significant difference between the two groups. Notably, after adding VG to both groups simultaneously, the number of mineralized nodules in the si SIRT4+VG group was significantly lower than in the negative control (hereafter denoted NC)+VG group. Figure S4I–K (Supporting Information) shows western blotting and the corresponding quantitative analysis for MC3T3‐E1 cells treated with VG and si SIRT4. In the process of inducing osteoblast differentiation, the expression of SIRT4 increased. The expression of SIRT4 decreased when it was used to transfect osteoblasts and the addition of VG significantly reversed the decrease in SIRT4. These results demonstrate that SIRT4 plays an essential role in promoting osteoblast differentiation.

### 2.7 Impact of Zn‐5Dy on HUVECs Angiogenesis

**Figure 6:**
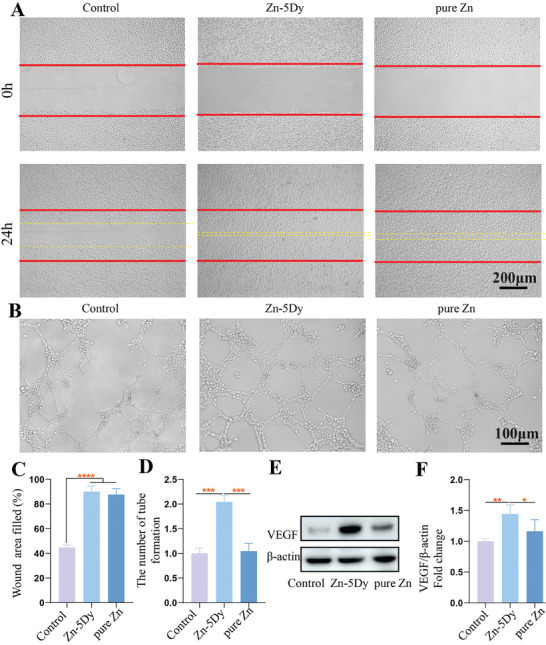
Cell migration and tube formation of HUVECs after culturing with 25% concentration extracts of Zn‐5Dy, pure Zn, and control for 24 h: A) cell‐migration images; B) tube‐formation capacity; C) quantitative wound‐healing areas; D) quantitative tube formation; E) protein levels of VEGF determined by western blotting; and F) quantitative western blotting after culturing with 25% concentration extracts of Zn‐5Dy, pure Zn, and control for 48 h. ^*^p < 0.05, ^**^p < 0.01, ^***^p < 0.001, ^****^p < 0.0001.

**Figure** **6**A shows cell‐migration images of HUVECs after culturing with 25% concentration extracts of Zn‐5Dy, pure Zn, and control for 24 h. The corresponding quantitative wound areas filled by HUVECs are shown in Figure 6C. Both the pure Zn and Zn‐5Dy extracts exhibit larger wound areas filled by HUVECs than the control group, indicating that the Zn‐containing samples exhibited good wound‐healing performance of HUVECs compared with the control group. In addition, the Zn‐5Dy alloy showed better performance in promoting wound healing than pure Zn. Figure 6B shows the tube formation of HUVECs induced by the extracts of Zn‐5Dy, Pure Zn, and control. The corresponding tube formations are shown in Figure 6D. The Zn‐5Dy extract shows the largest total tube length and largest number of tube branches among the three groups and the number of tube branches is two times that of the control group, revealing higher angiogenic ability. Figure 6E,F shows protein levels and quantitative western blotting for vascular endothelial growth factor (VEGF) expression of HUVECs after treatment with extracts of the Zn‐5Dy, pure Zn, and control for 48 h. The expression of SIRT4 in the Zn‐5Dy group is significantly higher than in the other two groups, indicating a better ability to promote angiogenesis. Overall, the Zn‐5Dy alloy significantly improves angiogenesis.

### 2.8 Influence of Zn‐5Dy on SIRT4‐Dependent Mitochondrial Function of HUVECs

**Figure 7:**
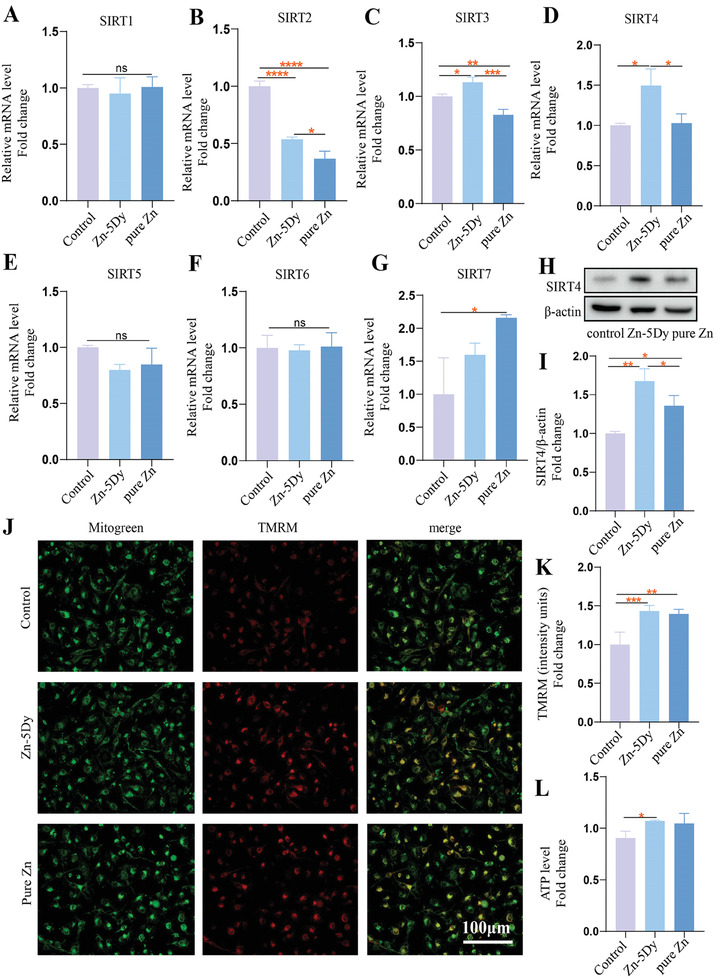
SIRT expression and mitochondrial function of HUVECs after culturing with 25% concentration extracts of Zn‐5Dy, pure Zn, and control for 48 h: A–G) gene expression level of SIRTs1–7; H) protein level of SIRT4 determined by western blotting; I) quantitative western blotting; J) representative images of TMRM staining; K) quantitative TMRM fluorescence; and L) ATP level in assigned groups. ^*^p < 0.05, ^**^p < 0.01, ^***^p < 0.001, ^****^p < 0.0001.

**Figure** **7**A–G shows the expression of SIRTs during the tube‐formation process of HUVECs after culturing with 25% concentration extracts of the Zn‐5Dy, pure Zn, and control for 48 h. Strikingly, the expression of SIRT3 and SIRT4 in the Zn‐5Dy group was higher than in both the pure Zn and control groups. Further, the expression of SIRT4 was raised by a higher multiple than that of SIRT3. Figure 7H,I shows the protein level of SIRT4 determined by western blotting, indicating that the Zn‐5Dy extract had a significantly greater effect on upregulating SIRT4 expression in comparison to the control and pure Zn groups. Figure 7J–L shows the mitochondrial function of HUVECs. It can be seen that the TMRM fluorescence and ATP levels of the Zn‐5Dy and the pure Zn groups are clearly higher than those of the control group, with a strong inference of increased mitochondrial function. Consequently, it can be concluded that Zn‐5Dy upregulates the SIRT4 expression and mitochondrial function of HUVECs.

### 2.9 Effect of Zn‐5Dy on HUVECs Angiogenesis via SIRT4

**Figure 8:**
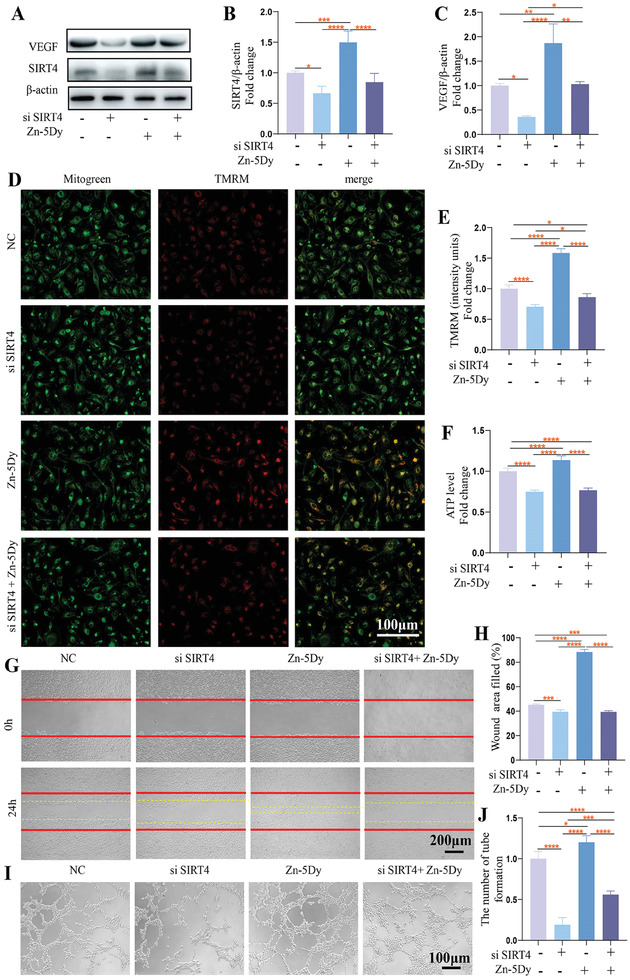
Cell migration and tube formation of HUVECs by regulating mitochondrial function through SIRT4 after pre‐transfection with si SIRT4 followed by treatment with 25% concentration extract of Zn‐5Dy: A) western blotting; B,C) quantitative western blotting; D) representative images of TMRM staining in assigned groups; E) quantitative TMRM; F) ATP levels in assigned groups; G) cell‐migration images; H) quantitative wound‐healing area; I) tube‐formation capacity; and J) quantitative tube formation. ^*^p < 0.05, ^**^p < 0.01, ^***^p < 0.001, ^****^p < 0.0001.

**Figure** **8**A–C shows western blotting and quantitative western blotting of HUVECs after pre‐transfection with si SIRT4 followed by treatment with 25% concentration extracts of the Zn‐5Dy, pure Zn, and control. The expression of VEGF was significantly inhibited by si SIRT4 transfection and the increase in VEGF expression mediated by the Zn‐5Dy extract was also inhibited. Figure 8D–F shows the mitochondrial function of HUVECs, indicating that the MMP and ATP levels were suppressed. The effect of the Zn‐5Dy extract on upregulating MMP and ATP levels was reversed by si SIRT4 transfection. Figure 8G,H shows migration rates of HUVECs and quantitative wound‐healing areas of the different groups. It can be seen that the migration rate is slower when treated with si SIRT4 and Zn‐5Dy extract than with Zn‐5Dy extract alone. Figure 8I,J shows tube formation and quantitative tube formation of the different groups. The Zn‐5Dy extract promoted tube formation, while si SIRT4 transfection reduced the number of tubes. Furthermore, the Zn‐5Dy extract did not contribute to tube formation when HUVECs were pre‐transfected with si SIRT4. Overall, the Zn‐5Dy significantly promotes the angiogenesis of HUVECs in a SIRT4‐dependent way.

### 2.10 Effect of SIRT4 on HUVECs Angiogenesis

Figure S5A–D (Supporting Information) shows the tube‐formation capacity and the corresponding quantitative analysis results. The number of tubes formed in the VEGF group was significantly higher than in the control group. The si SIRT4 group shows a significant decrease in the number of tubes compared with the control group and the addition of VEGF significantly reverses these results caused by si SIRT4 transfection. Figure S5E–H (Supporting Information) shows cell‐migration images and the corresponding quantitative analysis. Cells in the VEGF group migrate significantly faster than in the control group. Si SIRT4 transfection significantly reduced the migration rate of HUVECs and VEGF partially reversed the inhibitory effect of si SIRT4 on cell migration. Figure S5I–K (Supporting Information) shows western blotting of HUVECs treated with VEGF and si SIRT4. In the process of tube formation and cell migration, the expression of SIRT4 increased. The expression of SIRT4 decreased when si SIRT4 was used to transfect HUVECs and the addition of VEGF significantly reversed the decrease in SIRT4. These results suggest that SIRT4 also plays a role in promoting angiogenesis.

### 2.11 In Vivo Biosafety of Zn‐5Dy Alloy

Systematic studies are often necessary to assess the in vivo biosafety of biomaterials.^[^ ^31^ ^]^ Figure S6 (Supporting Information) shows histological sections of organs harvested from sacrificed rats, including the heart, liver, spleen, lung, and kidney. During 12 weeks following implantation, no evident pathogenic alterations were identified.

### 2.12 Impact of Zn‐5Dy Alloy on Angiogenesis and Bone Integration in Rats Through SIRT4

**Figure 9:**
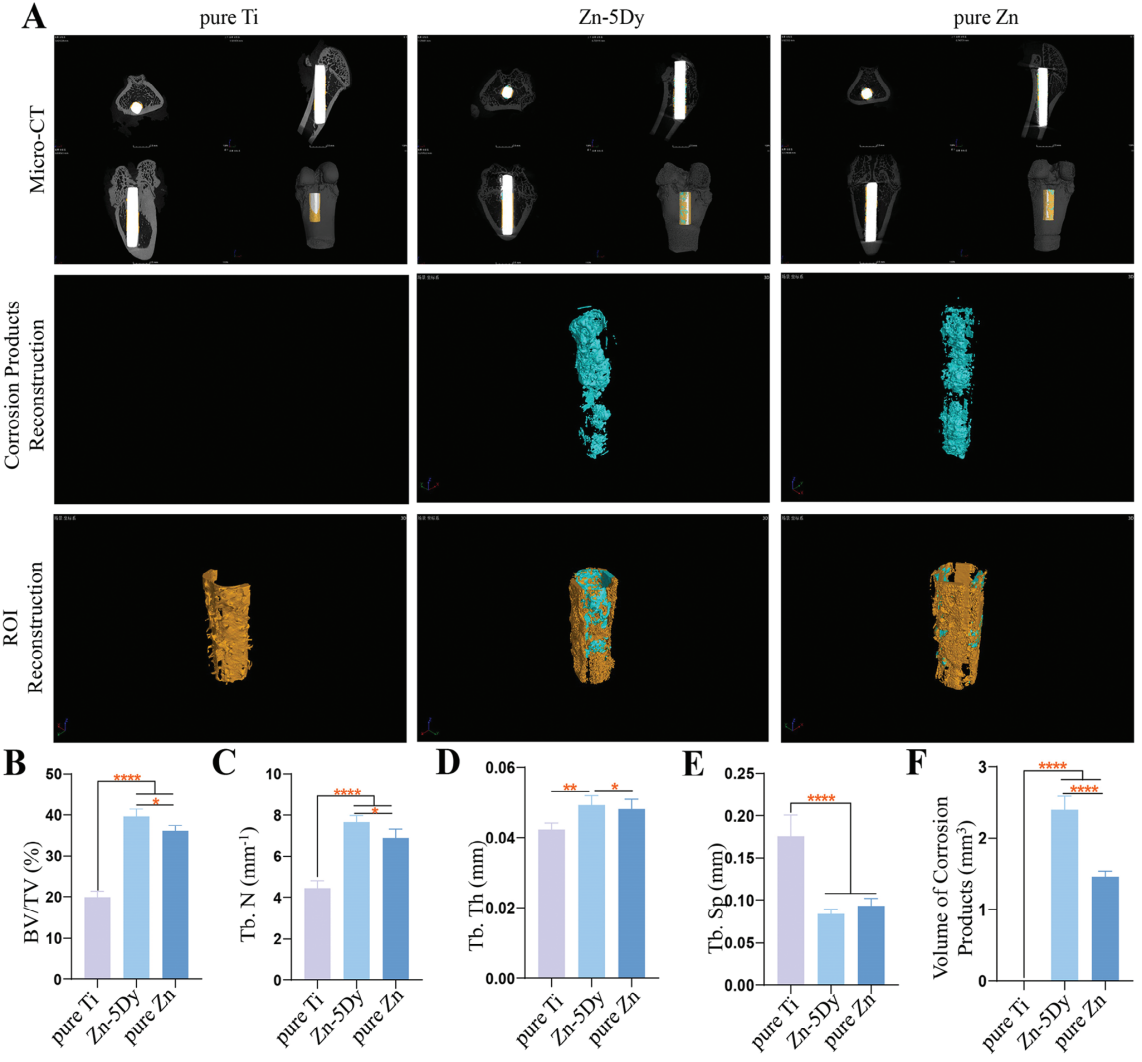
Osteointegration of pure Ti, Zn‐5Dy, and pure Zn after implantation in rat femurs for 12 weeks: A) micro‐CT images of implants and reconstructed images of implant corrosion products and newly formed bone; B–F) quantitative osteogenesis indices calculated from micro‐CT images. ^*^p < 0.05, ^**^p < 0.01, ^***^p < 0.001, ^****^p < 0.0001.

**Figure** **9**A shows micro‐computed tomography (micro‐CT) images of implants and reconstructed images of implant corrosion products and new bone in rat femurs after implantation of pure Ti, Zn‐5Dy, and pure Zn for 12 weeks. The reconstruction of the region of interest (ROI) surrounding the implants shows that there were more bone trabeculae on the surface of the ROI in the pure Zn and Zn‐5Dy groups than in the pure Ti group. Figure 9B–F shows the calculated bone volume fraction (BV/TV), trabecular number (Tb.N), trabecular thickness (Tb.Th), trabecular separation (Tb.Sp), and volume of corrosion products, respectively. After 12 weeks of implantation, BV/TV, Tb.N, and Tb.Th in the Zn‐5Dy and pure Zn groups were significantly higher than in the pure Ti group, indicating that the Zn‐containing samples showed a greater osteogenic property. Further, BV/TV, Tb.N, and Tb.Th in the Zn‐5Dy group were slightly higher than in the pure Zn group. There was no significant difference in the Tb.Sp value between Zn‐5Dy and pure Zn groups, but Tb.Sp in the pure Ti group was significantly higher than in the Zn‐5Dy and pure Zn groups. To visually display the degradation trend, the corrosion products of the pure Ti, Zn‐5Dy, and pure Zn implants were reconstructed (middle row of Figure 9A). The Zn‐5Dy group shows more corrosion products than the pure Zn group, while no corrosion products are observed in the pure Ti group, indicating that the degradation rate of the Zn‐5Dy was greater than that of pure Zn. Quantitative analysis also confirmed that the Zn‐5Dy degraded faster than pure Zn, consistent with the amounts of corrosion products shown in Figure 1C–F’ and the corrosion rates reported in a previous study.^[^ ^9^ ^]^

**Figure 10:**
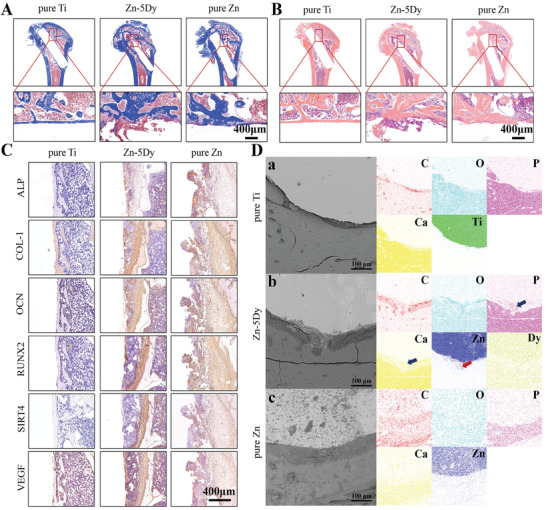
Osteointegration and angiogenesis of pure Ti, Zn‐5Dy, and pure Zn after implantation in rat femurs for 12 weeks: A,B) Masson and H&E staining of bone sections; C) immunohistochemical staining of ALP, COL‐1, OCN, RUNX2, SIRT4, and VEGF; and D) SEM images and EDS maps. ^*^p < 0.05, ^**^p < 0.01, ^***^p < 0.001, ^****^p < 0.0001.

**Figure** **10**A,B shows Masson and hematoxylin and eosin (H&E) staining images of rat femur bone sections after implantation of pure Ti, Zn‐5Dy, and pure Zn for 12 weeks. The boundary around the pure Ti implant is smooth with no new bone formation and only a small amount of collagen (stained blue). Similarly, only a small amount of new bone is observed around the pure Zn implant. In contrast, a great deal of new trabecular bone with a large amount of collagen and many osteocytes (whose cytoplasm is stained red) formed around the Zn‐5Dy implant, suggesting good integration between the implant and the host bone. The six critical immunohistochemical indices, i.e., ALP, COL‐1, OCN, RUNX2, SIRT4, and VEGF, are shown in Figure 10C. ALP is a vital marker for identifying osteoblast activity. COL‐1 is a main structural component of nascent bone formation. ALP, OCN, and COL‐1 are primarily expressed during osteoblast differentiation.^[^ ^8^ ^]^ Bruderer et al.^[^ ^32^ ^]^ reported that RUNX2 is effective in upregulating ALP, OCN, and COL‐1 expressions. In this study, the expressions of ALP, COL‐1, OCN, and RUNX2 in the pure Zn and Zn‐5Dy groups were higher than in the pure Ti group. There were more osteogenesis‐related genes in the Zn‐5Dy group than in the pure Zn group, except for ALP in the pure Zn group. Angiogenesis‐related gene VEGF and mitochondrial function‐related gene SIRT4 in the Zn‐5Dy group were significantly higher than in the pure Zn and pure Ti groups, indicating that the Zn‐5Dy had better osteogenic and angiogenic properties compared with the other two groups and regulated these functions through upregulating SIRT4 gene expression.

Figure 10D shows SEM images and EDS maps of cross‐sections of bone tissues and implants, indicating the distributions of C, oxygen, P, Ca, Dy, Ti, and Zn for the three groups of samples. The SEM images showed that the intramedullary implant remains intact as if it had just been implanted for the pure Ti group. The distribution of components suggests a clean and straight border between the intramedullary implant and the host bone tissue, indicating no interference exists between them. The boundary is broken for the pure Zn and Zn‐5Dy groups and there are many biodegradation products at their boundaries, suggesting that degradation of the pure Zn and Zn‐5Dy implants occurred. Further, the EDS maps for the Zn‐5Dy group show increased amounts of Ca and P, indicating increased bone mineral deposition (blue arrow) and accelerated bone growth. In addition, Zn is observed outside the bone–implant junction (red arrow), suggesting degradation and absorption of Zn in the Zn‐5Dy group. However, no significant mineralized matrix deposition or alloy degradation is seen in the pure Zn and pure Ti groups.

## 3 Discussion

**Figure 11:**
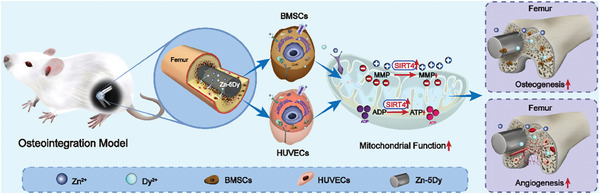
Illustration of the mechanism of a biodegradable Zn‐5Dy alloy modulating mitochondrial function and enhancing osteointegration and angiogenesis via upregulating SIRT4.

Bone fracture is a common clinical manifestation. Bone is the attachment point of muscle, so a bone fracture usually requires the implantation of a bone plate and bone nail to achieve rapid and efficient bone repair. Traditional metal materials, represented by titanium, are non‐degradable, prone to stress shielding, and often require revision operation to remove.^[^ ^3^ ^]^ Thus, degradable polymer materials came into playing. The degradation of polymer materials varies from several months to several years,^[^ ^33^ ^]^ accompanied by the production of acidic substances, often leading to local inflammation.^[^ ^34^ ^]^ In addition, the poor mechanical properties of polymer materials result in a larger size thickness, which limits its clinical application.^[^ ^35^ ^]^ The requirements of being degradable, having a smaller size and high strength make degradable alloys come into the public eye. Currently, biodegradable alloys that have been extensively studied in the field of orthopedic implants include magnesium (Mg), iron (Fe), and Zn alloys. The rapid degradation rate of Mg‐based materials leads to a sharp decline in mechanical properties, and the generation of excessive hydrogen gas alkalizes the local microenvironment, affecting tissue healing.^[^ ^36^ ^]^ Fe‐based alloys degrade slowly, and their ferromagnetism interferes with the radiological examination. Zn‐based alloys have degradation rates that better match the bone‐healing cycle than those of Fe‐ and Mg‐based alloys, making them more suitable as fracture‐fixation materials. A previous study^[^ ^9^ ^]^ indicated that Zn‐Dy alloys showed improved mechanical properties, suitable degradation rates, and enhanced in vitro biocompatibility. Zn alloys were also reported to play a catalytic role in osteointegration and angiogenesis^[^ ^37^, ^38^, ^39^ ^]^ however, the underlying mechanisms are still rarely reported. In this study, the Zn‐5Dy alloy is shown to have an upregulating mitochondrial function via SIRT4 (**Figure** **11**), contributing to osteointegration and angiogenesis, for the first time. This study demonstrates that the Zn‐5Dy alloy has biosafety, a degradation rate commensurate with the bone‐healing cycle, and satisfactory performance in relation to osteointegration and angiogenesis.

### 3.1 Mechanical Properties and Biodegradation

The HR Zn‐5Dy alloys showed significantly better mechanical properties (σ_uts_, σ_ys_, and ε) than those of pure Zn, mainly because the addition of the alloying element of Dy resulted in the formation of second‐phase DyZn_5_ at the grain boundaries,^[^ ^9^ ^]^ thereby playing roles in second‐phase strengthening and grain‐refining strengthening. Although the ε decreased with increasing Dy content in the Zn‐xDy (*x* = 1, 3, and 5 wt.%) alloys, the HR Zn‐5Dy alloy still showed an ε of 48.3%, significantly higher than the benchmark value of 15–18% required for bone‐fixation materials.^[^ ^40^ ^]^ With the continuous extension of immersion time in Hanks’ solution, the mechanical properties of the HR Zn–Dy alloy showed gradually decreasing tendencies. This is mainly because the surfaces of the tensile samples gradually corroded in the degradation process and formed relatively loose corrosion products, resulting in a decrease in the cross‐sectional areas of the samples, thereby leading to a decrease in strength. Also, as the recrystallization temperature of pure Zn is around room temperature (RT), the aging resistance of most Zn alloys is poor, resulting in the deterioration of the mechanical properties of the samples.^[^ ^41^ ^]^ The addition of Dy to Zn alloys can increase the recrystallization temperature and hinder the recrystallization process at RT,^[^ ^42^ ^]^ thus improving the aging resistance. The corrosion products of the HR samples were Zn_3_(PO_4_)_2_·4H_2_O, Zn_5_Cl_2_(OH)_8_·2H_2_O, Zn_5_(CO_3_)_2_(OH)_6_·H_2_O, Zn(OH)_2_, and hydroxyapatite during degradation and these can be absorbed and utilized by the body without causing a great burden or toxic effects. Nevertheless, the HR Zn‐5Dy after 60 d immersion still showed σ_uts_, σ_ys_, and ε values notably close to the required mechanical property benchmark values for biodegradable implant materials.^[^ ^40^ ^]^

### 3.2 In Vitro Osteogenic and Angiogenic Effects of Zn‐5Dy Alloy and Underlying Mechanisms

This study confirms that the Zn‐5Dy extract promoted angiogenesis and osteogenesis through co‐culturing with HUVECs and MC3T3‐E1 cells (Figures 6 and 3). However, there were no significant differences in osteogenic differentiation, endothelial cell migration, or mitochondrial function between the pure Zn and Zn‐5Dy groups. Based on these results, we can conclude that the osteoblast differentiation, endothelial cell migration, and mitochondrial function of the pure Zn and Zn‐5Dy groups reached plateaus, and it was challenging to distinguish the differences at the cellular level. The tube formation in the Zn‐5Dy group was significantly superior to that of the other two groups of pure Zn and control, which means that the extract of the Zn‐5Dy alloy contributed to fracture healing mainly by promoting tube formation. In addition, the osteogenic differentiation‐related genes, including RUNX2 and OCN, and angiogenesis‐related VEGF in the Zn‐5Dy group were significantly greater in number than in the pure Zn group. The protein expression of VEGF in HUVECs co‐cultured with the Zn‐5Dy extract was consistent with the gene expression of VEGF. Furthermore, the rare earth lanthanum has been reported to suppress arachidonic acid‐induced mitochondrial depolarization in PC12 cells.^[^ ^43^ ^]^ So, we assume two possible explanations for the above results: Dy^2+^ regulated the degradation of the Zn‐5Dy alloy, so the Zn^2+^ ion content in the extract increased. The other is that Dy^2+^ is involved in regulating mitochondrial function and promoting osteogenesis and angiogenesis in a way that is still unknown. Further investigation is needed to discover the source of this phenomenon.

Another interesting finding also attracted our attention: although the expressions of OCN, COL‐1, and RUNX2 genes in the Zn‐5Dy group were higher than in the pure Zn group, the expression of ALP was significantly lower than in the pure Zn group. These results are well explained by the fact that Zn^2+^ ions can stimulate exosomes produced by macrophages and thus upregulate the ALP activity of osteoblasts,^[^ ^44^ ^]^ an early marker of osteogenic differentiation.^[^ ^45^ ^]^ In addition, the results from this study demonstrate that the extract of the Zn‐5Dy alloy regulated the mitochondrial function of HUVECs and MC3T3‐E1 cells. Mitochondria are a major storage of Zn^2+^ ions,^[^ ^46^ ^]^ and the dysregulation of cellular Zn^2+^ homeostasis can generate mitochondrial stress.^[^ ^47^ ^]^ During the process of ZnO nanoparticle‐inducing apoptosis in human dermal fibroblasts, an increase in Zn^2+^ ion concentration resulted in the breakdown of mitochondrial membrane potential,^[^ ^48^ ^]^ contrary to the results from this study. This may be due to the different forms of the material. Compared with alloys, nanoparticles are more likely to release Zn^2+^ ions. A high concentration of Zn^2+^ ion destroys mitochondrial Zn^2+^ homeostasis.

SIRTs are NAD(+)‐dependent histone deacetylases involved in regulating osteoblast differentiation^[^ ^27^ ^]^ and endothelial function.^[^ ^30^ ^]^ In this study, we determined by real‐time polymerase chain reaction (RT‐PCR) that SIRT4 had the highest expression in HUVECs and MC3T3‐E1 cells treated with the Zn‐5Dy extract compared with other members of the SIRT family (Figures 4 and 7). As one of the three SIRTs localized in the mitochondrial matrix, SIRT4 is believed to respond to oxidative stress by regulating the activity of several mitochondrial proteins, thereby altering the cellular metabolic balance.^[^ ^49^ ^]^ SIRT4 knockdown decreased ATP production in dental papilla cells,^[^ ^50^ ^]^ and overexpression of SIRT4 mediated increased mitochondrial membrane potential in podocytes.^[^ ^51^ ^]^ Similarly, SIRT4 is involved in promoting osteointegration and angiogenesis of the Zn‐5Dy alloy by regulating mitochondrial function. In this study, we also confirm for the first time that the expression of SIRT4 increases during osteogenic differentiation and verify that SIRT4 increases during the angiogenesis of endothelial cells, indicating that SIRT4 promotes osteogenesis and angiogenesis in vitro. Additionally, the expression of dynamin‐related protein 1 (Drp1)^[^ ^52^ ^]^ and peroxisome proliferator‐activated receptor γ coactivator‐1α (PGC‐1α)^[^ ^53^ ^]^ increased in osteoblast differentiation. PGC‐1α,^[^ ^54^ ^]^ mitofusin 2,^[^ ^55^ ^]^ and putative kinase protein (PINK)^[^ ^56^ ^]^ also regulated angiogenesis. SIRTs can regulate the expression of PINK,^[^ ^57^ ^]^ PGC‐1α,^[^ ^26^ ^]^ and Drp1^[^ ^58^ ^]^ in some pathophysiological conditions. Therefore, SIRT4 may be closely related to mitochondrial autophagy, biogenesis, and dynamics in healing bone fractures. Further study is needed to explore this SIRT4‐dependent mitochondrial mechanism of the Zn‐5Dy alloy in promoting osteogenesis and angiogenesis.

### 3.3 In Vivo Osteogenic and Angiogenic Effects of Zn‐5Dy Alloy

In vivo animal experiments showed that the BV/TV, Tb.N, and Tb.Th of the new bone around the implant in the Zn‐5Dy group was higher than in the pure Zn and pure Ti groups. Immunohistochemical staining of the femur sections indicated that the expressions of COL‐1, OCN, RUNX2, and VEGF in the pure Zn group were significantly lower than in the Zn‐5Dy group, consistent with the above results, showing excellent osteogenesis and angiogenesis performance. However, the pure Zn group showed more significant positive results for ALP than the Zn‐5Dy group, which may be because the Zn^2+^ ion can boost the expression of ALP, an important marker of early osteogenesis.^[^ ^44^ ^]^ The SEM‐EDS results showed that many bone mineral deposition and degradation products were visible around the Zn‐5Dy implant. However, degradation products and mineralized matrix deposition around the pure Zn and Ti groups were hardly seen. This is mainly because the introduction of Dy accelerated the degradation of the Zn‐5Dy alloy, further promoting the increase in Zn^2+^ ion content around the implant, eventually leading to accelerated osteointegration. In summary, the Zn‐5Dy alloy has a more suitable degradation rate and better osteointegration and angiogenesis properties.

In addition, the SIRT4 expression around the Zn‐5Dy implant was much higher than in the pure Ti and Zn groups, corresponding to the effect of osseointegration, considering that the pure Zn group also had SIRT4 expression while the pure Ti group was completely negative. It can be inferred that the increased expression of SIRT4 may be related to the presence of Zn^2+^ ions in both the pure Zn and Zn‐5Dy groups, which is consistent with the in vitro results.

### 3.4 Limitation of the Current Study

Although we have confirmed that the Zn‐5Dy alloy has good biosafety and a degradation rate matching the bone‐healing cycle, and can regulate mitochondrial function through SIRT4 to promote angiogenesis and osteointegration both in vivo and in vitro, several limitations should be acknowledged:

First, we only conducted SIRT4 knockout in vitro cell models to confirm the important role of SIRT4 in regulating mitochondrial function in promoting angiogenesis and osteointegration by the Zn‐5Dy alloy. Our further study will build SIRT4 knockout mouse models for further validation in vivo.

Second, the regulating osteointegration and angiogenesis mechanism of Zn‐5Dy alloy should be studied by high‐throughput sequencing, and the existing mitochondria‐related indexes are insufficient to fully explain the mitochondrial molecular mechanism of the Zn‐5Dy alloy in promoting osteointegration and angiogenesis through SIRT4.

Third, in vivo experiments should be conducted on large animals, such as rabbits, sheep, and dogs, to simulate the interior environment of humans.

## 4 Conclusion

In this study, the Zn‐5Dy alloy was thoroughly investigated through mechanical, corrosion, in vitro, and in vivo tests. The key conclusions can be summarized as follows:

- The Zn‐5Dy alloy exhibits excellent mechanical properties, degradability, and biosafety in vitro and in vivo.
- SIRT4 regulates mitochondrial function as the Zn‐5Dy alloy promotes osteointegration and angiogenesis in vitro and in vivo.
- The Zn‐5Dy alloy demonstrates promising bone‐fracture healing performance both in vitro and in vivo.
- The Zn‐5Dy alloy is recommended for use in the internal fixation system for bone fractures in the form of bone nails and bone plates.

## 5 Experimental Section

### Material Preparation

The preparation process of the HR Zn‐1Dy, Zn‐3Dy, and Zn‐5Dy alloy plates with a final thickness of 1.5 mm was described in a previous work.^[^ ^9^ ^]^ Disc samples with a diameter of 8 mm and a thickness of 1.5 mm were cut from the HR plates using electrical discharge machining (EDM), ground with 2000‐grit SiC papers, and then ultrasonically cleaned with ethanol for immersion testing and the preparation of extracts for cytotoxicity, ALP staining, ARS staining, wound‐healing migration, tube formation, quantitative real‐time PCR analysis, western blotting, and ATP synthesis assays. HR pure Zn samples were prepared using the same process for comparison.

### Immersion Testing

The disc and tensile samples underwent immersion corrosion testing in Hanks' solution for 30 and 60 d at 37 ± 0.5 °C with a volume‐to‐area ratio of 20 mL cm^−2^.^[^ ^59^ ^]^ After 30 and 60 d of immersion, the morphologies and chemical compositions of the corrosion products on sample surfaces were examined using SEM (Pro X FEI, Phenom, Netherlands) combined with EDS (X‐Max, Oxford, UK) at 15 kV. The phase composition of the corrosion products on sample surfaces was identified by XRD (D/max 2500, Rigaku, Japan) with Cu–Kα radiation at a 2° min^−1^ scan rate in the 2*θ* range of 10°–80°.

### Tensile Properties

An Instron‐3369 universal testing system (Instron, MA, USA) with a displacement rate of 1 mm min^−1^ at RT was used to assess the tensile properties of the HR samples before and after immersion testing. According to ASTM E8/E 8M‐16,^[^ ^60^ ^]^ tensile samples in a plate shape with a 10 mm gauge length were cut using EDM parallel to the rolling direction and then ground with 2000‐grit SiC papers.

### Cell Culturing and Transfection

Cells used in this study were MC3T3‐E1 (ATCC, CRL‐2593, USA), HUVECs (ATCC, CRL‐1730, USA), BMSCs, and MG‐63 (ATCC, CRL‐1427TM, USA). MC3T3‐E1, BMSCs, MG‐63 cells, and HUVECs were respectively maintained in α‐modified minimal essential medium (α‐MEM; C12571500BT, Gibco, USA), Dulbecco's Modified Eagle Medium/Nutrient Mixture F‐12 (DMEM/F‐12; C11330500BT, Gibco, USA), Dulbecco's Modified Eagle Medium (DMEM/Sodium Pyruvate[−]; 11965‐092, Gibco, USA), and Dulbecco's Modified Eagle Medium (DMEM/Sodium Pyruvate[+]; C11995500BT, Gibco, USA) used in a humidified incubator with 5% CO_2_ and 95% air. All of these media were supplemented with 10% fetal bovine serum (04‐001‐1ACS, BI, Israel) and antibiotics (100 IU mL^−1^ penicillin G and 100 ng mL^−1^ streptomycin; 15140‐122, Gibco, USA). The metal extracts were prepared by immersing the disc samples into the four culture media with a volume‐to‐area ratio of 1.25 mL cm^−2^ for 3 d under the culturing conditions according to ISO 10993‐12.^[^ ^61^ ^]^ After digestion and decomposition, the Zn and Dy ion concentrations in the four extracts were measured using an inductively coupled plasma atomic emission spectrometer (720ES, Agilent, USA).

Small interfering RNA (siRNA) against the SIRT4 gene (Beijing, China) was provided by Tsingke Biotechnology Co., Ltd., Beijing, China. MC3T3‐E1 cells and HUVECs were cultured in 24‐well plates and transfected with 50 nM SIRT4 siRNA or negative control siRNA in serum‐free Opti‐MEM Reduced‐Serum Medium according to the manufacturer's procedure.^[^ ^62^ ^]^

### Cytotoxicity Testing

Cytotoxicity tests and cytoskeleton morphology were evaluated using MC3T3‐E1 cells, HUVECs, BMSCs, and MG‐63 cells according to ISO 10993–5.^[^ ^63^ ^]^ Cells were seeded into 96‐well plates at a density of 1 × 10^4^ cells per well. After 24 h, the culture medium in each well was replaced with 100 µL of 75%, 50%, and 25% sample extracts. After 1, 3, and 7 d of incubation, the medium was replaced with 100 µL of complete medium, and then 20 µL of MTT was added into each well. The MTT solution (M2128, Sigma, Germany) was withdrawn after 4 h of incubation in the cell incubator, and MTT formazan was dissolved in 100 µL dimethyl sulfoxide. The optical density value at 490 nm was measured using a microplate reader (M5, MD, USA). As a control, a flush cell culture medium was used.

Based on the results of the MTT tests, the sample extracts with 50% and 25% concentrations were used for live/dead and cytoskeletal staining via the abovementioned four cell models. The Calcein‐AM/PI Double Stain Kit (40747ES80, Yeasen, China) was used for the live/dead staining experiment. In brief, cells were seeded at a density of 1.5 × 10^4^ cells per well into 48‐well cell culture plates. After 24 h, the culture medium was replaced with 300 µL sample extract in each well and the plates were then incubated for 15 min at 37 °C with 2 µm Calcein‐AM and 1 µm propidium iodide in 1× assay buffer. Cytoskeletal staining was used to evaluate the cell morphology using 4′,6‐diamidino‐2‐phenylindole and fluorescein isothiocyanate (A12379, Thermo Fisher Scientific, USA). Fluorescence images were taken using a fluorescence microscope (Axio Observer3, Zeiss, Germany).

### ALP Staining

MC3T3‐E1 cells were seeded into 24‐well plates at a density of 2.5 × 10^4^ per well. After incubation for 24 h in the cell incubator and the cell fusion reaching 80%, the cell culture medium was replaced with media containing 25% extracts and supplemented with VG. During the incubation, the media were refreshed every 48 h. After 7 d of differentiation induction, the media were discarded and the plates were rinsed gently three times with PBS. Then, ALP staining was performed with a BCIP/NBT ALP color development kit (C3206, Beyotime, China), and the images were collected with a stereomicroscope (SMZ 800N, Nikon, Japan). Statistical analysis was performed using Image J software (version 1.80, National Institutes of Health, USA).

### Alizarin Red S (ARS) Staining and Quantitative Analysis

MC3T3‐E1 cells were seeded at a density of 2.5 × 10^4^ per well into 24‐well plates. Following 21 d of mineralization induction, the cells were gently washed and fixed for 30 min at 4 °C in a 4% paraformaldehyde (PFA) solution. Then 100 µL of ARS (G8550, Solarbio, China) solution was added to each well and the plates incubated for 1 h at RT. After PBS washing and image capture, 10% cetylpyridinium chloride (6004‐24‐6, Sigma, Germany) was added and the plates were incubated for 30 min, followed by absorbance measurement of the supernatant at 630 nm.

### Wound‐Healing Migration Assay

For the wound‐healing experiment, 3 × 10^5^ HUVECs were grown on 12‐well plates with various extracts. As the cells on the plates reached 90–95% confluence, scraping formed a straight line in the center of each well. After 0 and 24 h, photos were taken using a fluorescent microscope, and the area between the two edges of each scrape was computed using Image‐Pro Plus software (version 6.0, Media Cybernetics, USA).

### Tube Formation Assay

A 96‐well plate covered with Matrigel (354 234, Corning, USA) was used for the tube‐formation tests (354 234, Corning, USA), and a concentration of 5 × 10^4^ HUVECs per well was seeded into 0.2 mL of 25% extracts. Cells were incubated for 4 h at 37 °C before being photographed using a fluorescence microscope.

### Quantitative RT‐PCR Analysis

MC3T3‐E1 cells and HUVECs were cultivated under various conditions and quantitative RT‐PCR was utilized to assess gene expression. Total RNA was isolated from the two cells mentioned above using the Trizol reagent (15 596 026, Invitrogen, USA). The whole RNA was reverse‐transcribed to complementary DNA using a PrimeScript RT reagent kit (RR036A, Takara, Japan) according to the manufacturer's instructions; the RT‐PCR was carried out using a real‐time PCR system (SteponePlus, Thermo Fisher Scientific, USA) with a TB Green Premix (RR820A, Takara, Japan). The commercially synthesized primers (Tsingke Biotechnology Co., Ltd., China) are listed in **Table** **1**.

**Table 1:** Commercially synthesized primers.

Gene	Forward primer	Reverse primer
*M‐β‐actin*	5′TGCTGTCCCTGTATGCCTCTG3’	3′TGATGTCACGCACGATTTCC5’
*M‐ALP*	5′ACTCAGGGCAATGAGGTCAC3’	3′CACCCGAGTGGTAGTCACAA5’
*M‐RUNX2*	5′TTCAACGATCTGAGATTTGTGGG3’	3′GGATGAGGAATGCGCCCTA5’
*M‐COL‐1*	5′CCTAATGCTGCCTTTTCTGC3’	3′ATGTCCCAGCAGGATTTGAG5’
*M‐OCN*	5′AGACTCCGGCGCTACCTT3’	3′CTCGTCACAAGCAGGGTTAAG5’
*M‐SIRT1*	5′GTTGACCGATGGACTCCTCAC3’	3′AGCTCAGGTGGAGGAATTGT5’
*M‐SIRT2*	5′AGCCAACCATCTGCCACTAC3’	3′ATGTGTAGAAGGTGCCGTGG5’
*M‐SIRT3*	5′GGCACTACAGGCCCAATGT3’	3′CTCTCAAGCCCGTCGATGT5’
*M‐SIRT4*	5′GGCGACGTGTTCCTCACTG3’	3′ACAAAGTCAACCTTGTCTGGG5’
*M‐SIRT5*	5′GTCATCACCCAGAACATCGA3’	3′ACGTGAGGTCGCAGCAAGCC5’
*M‐SIRT6*	5′CTCCAGCGTGGTTTTCCACA3’	3′GCCCATGCGTTCTAGCTGA5’
*M‐SIRT7*	5′CTAAGCGAAGCGGAGCCTAC3’	3′GTGGAGCCCATCACAGTTCT5’
*H‐β‐actin*	5′GAGCACAGAGCCTCGCCTTTGCC3’	3′CGAGCGCGGCGATATCATCATCC5’
*H‐SIRT1*	5′GGAGCAGATTAGTAGGCGGC3’	3′ACCTCAGCGCCATGGAAAAT5’
*H‐SIRT2*	5′TCCTGCGGAACTTATTCTCCC3’	3′GATGGTTGGCTTGAACTGCC5’
*H‐SIRT3*	5′CCCAGTGGCATTCCAGACTT3’	3′AAGGGCTTGGGGTTGTGAAA5’
*H‐SIRT4*	5′CTCGAAAGCCTCCATTGGGT3’	3′GGCCAGCCTACGAAGTTTCT5’
*H‐SIRT5*	5′AGGAAAAGGGTGTGAAGAGGC3’	3′GGAAGTGCCCACCACTAGAC5’
*H‐SIRT6*	5′ACGCAGTACGTCAGAGACAC3’	3′GTTGACAATGACCAGACGGC5’
*H‐SIRT7*	5′CTTGGTCGTCTACACAGGCG3’	3′GGTGATGCTCATGTGGGTGA5’

### Western Blotting

The protein expression levels were measured using the western blotting technique. HUVECs and MC3T3‐E1 cells were grown under varied conditions before being collected and homogenized in a cell lysis buffer (9803, Cell Signaling Technology, USA). The Bradford Protein Assay Kit (23 236, Thermo Fisher Scientific, USA) was used to quantify protein quantities in cell lysates. Next, proteins in whole‐cell lysates were separated using sodium dodecyl sulfate‐polyacrylamide gel electrophoresis on a 12% gel and transferred onto polyvinylidene difluoride (PVDF) membranes (456‐1094, Bio‐Rad, USA). The PVDF membranes were blocked with 5% nonfat dry milk diluted in Tris‐buffered saline (pH 7.4) containing 0.05% Tween‐20 (TBST) for 90 min at RT. Then the membranes were washed twice with TBST (3 min each wash) and incubated overnight at 4 °C with the specified primary antibodies against VEGF (19003‐1‐AP, Proteintech, USA; 1:1000) and SIRT4 (69786S, Cell Signaling Technology, USA; 1:1000). Subsequently, the membranes were washed three times with TBST (5 min each wash) and incubated with the anti‐mouse or anti‐rabbit secondary antibody (1:4000) in 5% nonfat dry milk diluted in TBST for 60 min at RT. Finally, the membranes were washed three times with TBST for 5 min and the protein bands were detected using an enhanced chemiluminescence detection kit (1 251 473, Thermo Fisher Scientific, USA). An imaging system (ChemiDoc Touch Imaging System, version 5.1, Bio‐Rad, USA) was used for quantitative densitometric analysis of the detected protein bands and quantification was conducted using Image J software (version 1.80, National Institutes of Health, USA).

### Measurement of MMP

MC3T3‐E1 cells were seeded into 48‐well plates at a density of 9 × 10^3^ cells per well and were cultured with 25% sample exacts. As a unique fluorogenic dye, TMRM (T668, Invitrogen, USA) was employed to detect MMP in living cells. For 30 min at 37 °C, the cells were treated in new culture media with 100 nM MT Green (M7514, Invitrogen, USA) and 100 nM TMRM. A fluorescent microscope was used to collect images by a technician who was blind to the treatments. Excitation wavelengths of 543 and 488 nm were employed for TMRM and MT Green, respectively. All images were processed using Image J software (version 1.80, National Institutes of Health, USA).

### ATP Synthesis Assay

Whole‐cell lysates were produced for assessing ATP levels by lysing the cells in a lysis solution included in the ATP assay kit (A22066, Invitrogen, USA). Following 5 min of centrifugation at 12 000 rpm at 4 °C, the supernatants were transferred to a fresh 1.5 mL tube for ATP production analysis. A microplate reader (M5, MD, USA) and 100 µL ATP detection buffer were used to measure the luminescence of a 100 µL sample. With a known quantity, a standard curve of ATP concentrations (1 nM–1 µm) was created. All ATP studies were carried out according to the methods described in the previous study.^[^ ^64^ ^]^

### In Vivo Bone Osteointegration Effect of Zn‐5Dy Alloy

Based on the preceding in vitro research findings, Zn‐5Dy was chosen as a candidate for further in vivo enquiry. Pure Zn and Zn‐5Dy were produced as intramedullary nails in cylindrical rods (Φ 1.5 mm × 8 mm) with pointed ends to enable implantation. For comparison, pure Ti with the same shape was set as the control group. Fifteen male Sprague–Dawley rats aged 8 weeks old were randomly divided into three groups. The pure Ti group was taken as the negative control, the pure Zn group as the positive control, and the remaining group was implanted with the Zn‐5Dy implants. The animals were kept in transparent plastic cages with clean bedding, free access to water, and standard laboratory food. Pentobarbital sodium (40 mg kg^−1^) was administered intraperitoneally to anesthetize the rats before surgery. The right hind leg of each rat was fixated, shaved, and depilated. For surgical implantation, the lateral approach to the knee joint was used. Metals were implanted by pre‐drilling a femur medullary cavity with a 2 mm electric drill, and each rat was implanted with one type of sample. The incision was then meticulously sutured layer by layer and appropriately treated. All surgical procedures were carried out under aseptic circumstances. Each rat was given prophylactic antibiotics 3 d after surgery to prevent infection.

The creation of new bone and the corrosion products of rods were scanned using a high‐resolution micro‐CT scanner for harvested femoral samples. The scanning resolution was ≈9.38 µm at 160 kV and 160 A. The scanning matrix size was 1024 × 1024. At the implantation site, ≈500 layers were continually scanned. The bone‐volume percentage within an ROI known as BV/TV was calculated using Volume Graphics Studio MAX software (version 2.1, Volume Graphics GmbH, Germany). Multimodal 3D Visualization (Siemens, Germany) software was used to build three‐dimensional images of each sample.

The femur preserved in 4% PFA solution was decalcified for 8 weeks in 0.5 mol L^−1^ ethylenediamine tetraacetic acid (pH 7.3) before being embedded in paraffin. H&E and Masson staining were used to stain thin sections of 5 µm thickness. After deparaffinization and rehydration, additional sections were utilized for immunohistochemistry staining. Anti‐ALP (ab224335, Abcam, USA, 1:200), anti‐OCN (39382S, CST, USA, 1:200), anti‐COL‐1 (72026S, CST, USA, 1:200), anti‐RUNX2 (sc‐390351, Santa Cruz Biotechnology, USA, 1:800), anti‐SIRT4 (66543‐1, Proteintech, USA, 1:500), and anti‐VEGF (19003‐1‐AP, Proteintech, USA, 1:500) were used to stain the sections for immunohistochemistry. The sections were colored with 3‐3′‐diaminobenzidine tetrahydrochloride before being counterstained with Mayer's hematoxylin (G1080, Solarbio, China). Histological images were all obtained using a scanner (Pannoramic MIDI, 3DHISTECH, Hungary).

Femoral/rod constructs from each group were fixed in 4% PFA solution, dried with gradient ethanol, and embedded in polymethylmethacrylate for histological inspection. Then a saw microtome (EXAKT Apparatebau, Norderstedt, Hamburg, Germany) was used to cut thick sections of 150–200 µm, ground, and polished to a final thickness of roughly 50 µm. The morphology and chemical composition of bone sample sections were observed and analyzed using SEM and EDS after polishing and sputtering with platinum. The implants were defined as areas with overlapping elemental Ti, Zn, and Dy signals on EDS maps and the interior bone tissues as areas with overlapping elemental Ca and P signals.

At each time point, patches of tissue from the heart, liver, lung, spleen, and kidney organs were removed in addition to bone. Histological examination of the soft tissues was performed using a scanner after fixing, dehydrating, embedding in paraffin, and H&E staining.

### Statistical Analysis

All data were presented as mean ± standard deviation (SD) from triplicate experiments. Statistical analysis was performed using one‐way analysis of variance (ANOVA) followed by Tukey's post hoc test via GraphPad Prism 8.0 software (GraphPad Prism Software Inc., San Diego, CA, USA). Statistical significance was confirmed when the *p*‐value < 0.05.

## Conflict of Interest

The authors declare no conflict of interest.

## Supporting information

Supporting Information

## Data Availability

Research data are not shared.

## References

[1] H. Wei , J. Cui , K. Lin , J. Xie , X. Wang , Bone Res. 2022, 10, 17. 35197462 10.1038/s41413-021-00180-y PMC8866424

[2] Y. Zhang , W. Ma , Y. Zhan , C. Mao , X. Shao , X. Xie , X. Wei , Y. Lin , Bone Res. 2018, 6, 37. 30603226 10.1038/s41413-018-0042-7 PMC6306486

[3] Y. Zhang , J. Xu , Y. C. Ruan , M. K. Yu , M. O'laughlin , H. Wise , D. Chen , L. Tian , D. Shi , J. Wang , S. Chen , J. Q. Feng , D. H. K. Chow , X. Xie , L. Zheng , L. Huang , S. Huang , K. Leung , N. Lu , L. Zhao , H. Li , D. Zhao , X. Guo , K. Chan , F. Witte , H. C. Chan , Y. Zheng , L. Qin , Nat. Med. 2016, 22, 1160. 27571347 10.1038/nm.4162 PMC5293535

[4] X. Tong , H. Wang , L. Zhu , Y. Han , K. Wang , Y. Li , J. Ma , J. Lin , C. Wen , S. Huang , Acta Biomater. 2022, 146, 478. 35580830 10.1016/j.actbio.2022.05.017

[5] R. Marchan , C. Cadenas , H. M. Bolt , Arch. Toxicol. 2012, 86, 519. 22415766 10.1007/s00204-012-0843-1

[6] S. Wang , R. Gu , F. Wang , X. Zhao , F. Yang , Y. Xu , F. Yan , Y. Zhu , D. Xia , Y. Liu , Mater. Today Bio 2022, 13, 100202. 10.1016/j.mtbio.2021.100202 PMC8753274 35036897

[7] D. Meng , L. Dong , Y. Yuan , Q. Jiang , Regener. Biomater. 2019, 6, 13. 10.1093/rb/rby027 PMC6362821 30740238

[8] Z. Zhang , B. Jia , H. Yang , Y. Han , Q. Wu , K. Dai , Y. Zheng , Biomaterials 2021, 275, 120905. 34087587 10.1016/j.biomaterials.2021.120905

[9] X. Tong , Y. Han , R. Zhou , W. Jiang , L. Zhu , Y. Li , S. Huang , J. Ma , C. Wen , J. Lin , Acta Biomater. 2023, 155, 684. 36328128 10.1016/j.actbio.2022.10.053

[10] Z. Li , S. Liu , T. Fu , Y. Peng , J. Zhang , Stem Cell Res. Ther. 2019, 10, 351. 31775910 10.1186/s13287-019-1441-4 PMC6880487

[11] S. Maria , R. M. Samsonraj , F. Munmun , J. Glas , M. Silvestros , M. P. Kotlarczyk , R. Rylands , A. Dudakovic , A. J. Van Wijnen , L. T. Enderby , H. Lassila , B. Dodda , V. L. Davis , J. Balk , M. Burow , B. A. Bunnell , P. A. Witt‐Enderby , J. Pineal Res. 2018, 64, e12465. 10.1111/jpi.12465 PMC6711668 29285799

[12] A. Longchamp , T. Mirabella , A. Arduini , M. R. Macarthur , A. Das , J. H. Treviño‐Villarreal , C. Hine , I. Ben‐Sahra , N. H. Knudsen , L. E. Brace , J. Reynolds , P. Mejia , M. Tao , G. Sharma , R. Wang , J.‐M. Corpataux , J.‐A. Haefliger , K. H. Ahn , C.‐H. Lee , B. D. Manning , D. A. Sinclair , C. S. Chen , C. K. Ozaki , J. R. Mitchell , Cell 2018, 173, 117. 29570992 10.1016/j.cell.2018.03.001 PMC5901681

[13] H. Mannell , P. Kameritsch , H. Beck , A. Pfeifer , U. Pohl , K. Pogoda , Int. J. Mol. Sci. 2021, 23, 294. 35008716 10.3390/ijms23010294 PMC8745637

[14] A. P. Kusumbe , S. K. Ramasamy , R. H. Adams , Nature 2014, 513, 574.

[15] S. K. Ramasamy , A. P. Kusumbe , L. Wang , R. H. Adams , Nature 2014, 507, 376. 24647000 10.1038/nature13146 PMC4943529

[16] T. Xu , Y. Yang , D. Suo , H. P. Bei , X. Xu , X. Zhao , Small 2022, 18, 2205695. 10.1002/smll.202200314 35261154

[17] L. Zhang , G. Jiao , S. Ren , X. Zhang , C. Li , W. Wu , H. Wang , H. Liu , H. Zhou , Y. Chen , Stem Cell Res. Ther. 2020, 11, 38. 31992369 10.1186/s13287-020-1562-9 PMC6986095

[18] W. Zhang , X. Lu , Z. Yuan , M. Shen , Y. Song , H. Liu , J. Deng , X. Zhong , X. Zhang , Int. J. Nanomed. 2019, 14, 977. 10.2147/IJN.S190766 PMC6368129 30787611

[19] H. Zhang , R. Xu , B. Li , Z. Xin , Z. Ling , W. Zhu , X. Li , P. Zhang , Y. Fu , J. Chen , L. Liu , J. Cheng , H. Jiang , Cell Death Differ. 2021, 29, 351. 34497381 10.1038/s41418-021-00858-0 PMC8816946

[20] L.‐T. Wang , P.‐C. He , A.‐Q. Li , K.‐X. Cao , J.‐W. Yan , S. Guo , L. Jiang , L. Yao , X.‐Y. Dai , D. Feng , Y.‐M. Xu , N. Tan , Acta Pharmacol. Sin. 2021, 42, 2033. 33664417 10.1038/s41401-021-00623-6 PMC8632980

[21] X. Wang , Z. Chen , J. Xu , S. Tang , N. An , L. Jiang , Y. Zhang , S. Zhang , Q. Zhang , Y. Shen , S. Chen , X. Lan , T. Wang , L. Zhai , S. Cao , S. Guo , Y. Liu , A. Bi , Y. Chen , X. Gai , Y. Duan , Y. Zheng , Y. Fu , Y. Li , L. Yuan , L. Tong , K. Mo , M. Wang , S.‐H. Lin , M. Tan , et al., Cell Res. 2022, 32, 638. 35459936 10.1038/s41422-022-00650-w PMC9253147

[22] Q. Li , Z. Gao , Y. Chen , M.‐X. Guan , Protein Cell 2017, 8, 439. 28271444 10.1007/s13238-017-0385-7 PMC5445026

[23] S. Herkenne , O. Ek , M. Zamberlan , A. Pellattiero , M. Chergova , I. Chivite , E. Novotná , G. Rigoni , T. B. Fonseca , D. Samardzic , A. Agnellini , C. Bean , G. Di Benedetto , N. Tiso , F. Argenton , A. Viola , M. E. Soriano , M. Giacomello , E. Ziviani , G. Sales , M. Claret , M. Graupera , L. Scorrano , Cell Metab. 2020, 31, 987. 32315597 10.1016/j.cmet.2020.04.007

[24] Y. Kida , M. S. Goligorsky , Can. J. Cardiol. 2016, 32, 634. 26948035 10.1016/j.cjca.2015.11.022 PMC4848124

[25] A. Zullo , R. Guida , R. Sciarrillo , F. P. Mancini , Front. Endocrinol. 2022, 13, 858330. 10.3389/fendo.2022.858330 PMC8971707 35370975

[26] C. K. Singh , G. Chhabra , M. A. Ndiaye , L. M. Garcia‐Peterson , N. J. Mack , N. Ahmad , Antioxid. Redox Signaling 2018, 28, 643. 10.1089/ars.2017.7290 PMC5824489 28891317

[27] C. Buccoliero , M. Dicarlo , P. Pignataro , F. Gaccione , S. Colucci , G. Colaianni , M. Grano , Int. J. Mol. Sci. 2021, 22, 4670. 33925111 10.3390/ijms22094670 PMC8124835

[28] H. Chen , J. Kang , F. Zhang , T. Yan , W. Fan , H. He , F. Huang , Int. J. Biochem. Cell Biol. 2021, 134, 105962. 33636397 10.1016/j.biocel.2021.105962

[29] Y. Tao , S. Yu , M. Chao , Y. Wang , J. Xiong , H. Lai , Mol. Med. Rep. 2019, 19, 4973. 31059091 10.3892/mmr.2019.10161

[30] S. J. Pecher , A. B. Potthast , F. Von Versen‐Höynck , A. M. Das , J. Clin. Med. 2020, 9, 2604. 32796661 10.3390/jcm9082604 PMC7464651

[31] I. C. Santos , G. S. Gazelle , L. A. Rocha , J. M. R. Tavares , Expert Rev. Med. Devices 2012, 9, 299. 22702261 10.1586/erd.12.3

[32] M. Bruderer , R. Richards , M. Alini , M. Stoddart , Eur. Cells Mater. 2014, 28, 269. 10.22203/ecm.v028a19 25340806

[33] S. Farah , D. G. Anderson , R. Langer , Adv. Drug Delivery Rev. 2016, 107, 367. 10.1016/j.addr.2016.06.012 27356150

[34] O. Böstman , H. Pihlajamäki , Biomaterials 2000, 21, 2615. 11071611 10.1016/s0142-9612(00)00129-0

[35] R. Naseem , C. Tzivelekis , M. J. German , P. Gentile , A. M. Ferreira , K. Dalgarno , Molecules 2021, 26, 992. 33668466 10.3390/molecules26040992 PMC7917714

[36] D. C. Martinez , A. Dobkowska , R. Marek , H. Ćwieka , J. Jaroszewicz , T. Płociński , W. Święszkowski , Bioact. Mater. 2023, 28, 132. 37250863 10.1016/j.bioactmat.2023.05.004 PMC10209338

[37] B. Jia , H. Yang , Z. Zhang , X. Qu , X. Jia , Q. Wu , Y. Han , Y. Zheng , K. Dai , Bioact. Mater. 2021, 6, 1588. 33294736 10.1016/j.bioactmat.2020.11.007 PMC7691683

[38] M. Toledano , M. Vallecillo‐Rivas , M. T. Osorio , E. Muñoz‐Soto , M. Toledano‐Osorio , C. Vallecillo , R. Toledano , C. D. Lynch , M.‐A. Serrera‐Figallo , R. Osorio , Polymers 2021, 13, 1797. 34072433 10.3390/polym13111797 PMC8199215

[39] Z. Wang , W. Wang , X. Zhang , F. Cao , T. Zhang , D. Bhakta Pokharel , D. Chen , J. Li , J. Yang , C. Xiao , Y. Ren , G. Qin , D. Zhao , Materials 2022, 15, 7117. 36295204 10.3390/ma15207117 PMC9608845

[40] P. K. Bowen , J. Drelich , J. Goldman , Adv. Mater. 2013, 25, 2577. 23495090 10.1002/adma.201300226

[41] M. S. Ardakani , E. Mostaed , M. Sikora‐Jasinska , S. L. Kampe , J. W. Drelich , Mater. Sci. Eng. A 2020, 770, 138529. 10.1016/j.msea.2019.138529 PMC7450801 32863579

[42] K. K. Deng , X. J. Wang , M. Y. Zheng , K. Wu , Mater. Sci. Eng. A 2013, 560, 824.

[43] N. Doroshenko , P. Doroshenko , Eur. J. Pharmacol. 2007, 567, 36. 17499712 10.1016/j.ejphar.2007.04.019

[44] J. Liu , Y. Zhao , Y. Zhang , X. Yao , R. Hang , J. Mater. Chem. B 2021, 9, 3800. 33899897 10.1039/d1tb00112d

[45] N. Li , M. Yan , Y. Chen , Y. Wang , J. Wu , L. Fu , J. Yu , Exp. Cell Res. 2021, 407, 112780. 34411610 10.1016/j.yexcr.2021.112780

[46] H. Deng , X. Qiao , T. Xie , W. Fu , H. Li , Y. Zhao , M. Guo , Y. Feng , L. Chen , Y. Zhao , L. Miao , C. Chen , K. Shen , X. Wang , Proc. Natl. Acad. Sci. USA 2021, 118, e2023909118. 34433664 10.1073/pnas.2023909118 PMC8536367

[47] S. A. Dabravolski , N. K. Sadykhov , A. G. Kartuesov , E. E. Borisov , V. N. Sukhorukov , A. N. Orekhov , Int. J. Mol. Sci. 2022, 23, 6890.35805904

[48] K. Meyer , P. Rajanahalli , M. Ahamed , J. J. Rowe , Y. Hong , Toxicol. In Vitro 2011, 25, 1721. 21903158 10.1016/j.tiv.2011.08.011

[49] C. Carrico , J. G. Meyer , W. He , B. W. Gibson , E. Verdin , Cell Metab. 2018, 27, 497. 29514063 10.1016/j.cmet.2018.01.016 PMC5863732

[50] Y.‐E. Jang , S.‐H. Go , B.‐N. Lee , H.‐S. Chang , I.‐N. Hwang , W.‐M. Oh , Y.‐C. Hwang , Restor. Dent. Endod. 2015, 40, 223. 26295026 10.5395/rde.2015.40.3.223 PMC4534727

[51] J.‐X. Shi , Q.‐J. Wang , H. Li , Q. Huang , Exp. Ther. Med. 2016, 13, 342. 28123512 10.3892/etm.2016.3938 PMC5245066

[52] X. Zhou , S.‐N. Xu , S.‐T. Yuan , X. Lei , X. Sun , L. Xing , H.‐J. Li , C.‐X. He , W. Qin , D. Zhao , P.‐Q. Li , E. Moharomd , X. Xu , H.‐L. Cao , Cell Biosci. 2021, 11, 159. 34399835 10.1186/s13578-021-00639-9 PMC8369777

[53] B. Yu , L. Huo , Y. Liu , P. Deng , J. Szymanski , J. Li , X. Luo , C. Hong , J. Lin , C.‐Y. Wang , Cell Stem Cell 2018, 23, 193. 30017591 10.1016/j.stem.2018.06.009 PMC6322535

[54] Z. Arany , S.‐Y. Foo , Y. Ma , J. L. Ruas , A. Bommi‐Reddy , G. Girnun , M. Cooper , D. Laznik , J. Chinsomboon , S. M. Rangwala , K. H. Baek , A. Rosenzweig , B. M. Spiegelman , Nature 2008, 451, 1008. 18288196 10.1038/nature06613

[55] J. J. Lugus , G. A. Ngoh , M. M. Bachschmid , K. Walsh , J. Mol. Cell. Cardiol. 2011, 51, 885. 21839087 10.1016/j.yjmcc.2011.07.023 PMC3208756

[56] R. J. Youle , D. P. Narendra , Nat. Rev. Mol. Cell Biol. 2010, 12, 9. 10.1038/nrm3028 PMC4780047 21179058

[57] L. P. Poole , K. F. Macleod , Cell. Mol. Life Sci. 2021, 78, 3817. 33580835 10.1007/s00018-021-03774-1 PMC8259496

[58] G. López‐Lluch , Mech. Ageing Dev. 2017, 162, 108. 27993601 10.1016/j.mad.2016.12.005

[59] F. Witte , F. Feyerabend , P. Maier , J. Fischer , M. Störmer , C. Blawert , W. Dietzel , N. Hort , Biomaterials 2007, 28, 2163. 17276507 10.1016/j.biomaterials.2006.12.027

[60] ASTM E8/E 8M–16, Standard Test Methods for Tension Testing of Metallic Materials , ASTM International, West Conshohocken, PA, 2011.

[61] ISO 10993‐12, Biological evaluation of medical devices. Part 12: sample preparation and reference materials, in, International Organisation for Standardization, Geneva, Switzerland, 2021.

[62] Y. Yang , Y. Lin , M. Wang , K. Yuan , Q. Wang , P. Mu , J. Du , Z. Yu , S. Yang , K. Huang , Y. Wang , H. Li , T. Tang , Bone Res. 2022, 10, 26. 35260560 10.1038/s41413-022-00198-w PMC8904790

[63] ISO 10993–5, Biological evaluation of medical devices. Part 5: tests for in vitro cytotoxicity, in, International Organisation for Standardization, Geneva, Switzerland, 2009.

[64] Y. X. Mao , W. J. Cai , X. Y. Sun , P. P. Dai , X. M. Li , Q. Wang , X. L. Huang , B. He , P. P. Wang , G. Wu , J. F. Ma , S. B. Huang , Cell Death Dis. 2018, 9, 674. 29867140 10.1038/s41419-018-0718-3 PMC5986782

