# Supplementary material for: Biodegradable Zn‐5Dy Alloy with Enhanced Osteo/Angio‐Genic Activity and Osteointegration Effect via Regulation of SIRT4‐Dependent Mitochondrial Function
Source: Adv Sci (Weinh). 2024 Jan 19;11(13):2307812. doi: 10.1002/advs.202307812 (PMC10987155; doi:10.1002/advs.202307812)
Supplement: Supplementary file 1 — Supporting Information [file ADVS-11-2307812-s001.pdf]

ADVANCED SCIENCE
Open Access

# Supporting Information

for *Adv. Sci.*, DOI 10.1002/advs.202307812

Biodegradable Zn-5Dy Alloy with Enhanced Osteo/Angio-Genic Activity and Osteointegration Effect via Regulation of SIRT4-Dependent Mitochondrial Function

*Yue Han, Xian Tong, Runqi Zhou, Yilin Wang, Yuge Chen, Liang Chen, Xinhua Hong, Linmei Wu, Zhiqiang Lin, Yichi Zhang, Xuejia Zhang, Chaoming Hu, Bin Li, Yifan Ping, Zelin Cao, Zhou Ye, Zhongchen Song, Yuncang Li, Cuie Wen*, Yongsheng Zhou*, Jixing Lin* and Shengbin Huang**

# Biodegradable Zn-5Dy alloy with enhanced osteo/angio-genic activity and osteointegration effect via regulation of SIRT4-dependent mitochondrial function

Yue Han, Xian Tong, Runqi Zhou, Yilin Wang, Yuge Chen, Liang Chen, Xinhua Hong, Linmei Wu, Zhiqiang Lin, Yichi Zhang, Xuejia Zhang, Chaoming Hu, Bin Li, Yifan Ping, Zelin Cao, Zhou Ye, Zhongchen Song, Yuncang Li, Cuie Wen*, Yongsheng Zhou*, Jixing Lin*, and Shengbin Huang*

Y. Han, Dr. X. Tong, R.Q. Zhou, Y.L. Wang, Y.G. Chen, L. Chen, X.H. Hong, L.M. Wu, Z.Q. Lin, Y.C. Zhang, X.J. Zhang, C.M. Hu, B. Li, Y.F. Ping, Z.L. Cao, Dr. J.X. Lin, Prof. Dr. S.B. Huang

Institute of Stomatology, School and Hospital of Stomatology

Wenzhou Medical University

Wenzhou 325027, China

E-mail: jixing.lin@wmu.edu.cn (J.X. Lin); huangsb003@wmu.edu.cn (S.B. Huang).

Y.G. Chen

Department of Dentistry, Faculty of Medicine and Dentistry

University of Alberta

Edmonton, T6G2R3, Canada

Prof. Dr. Z. Ye

Applied Oral Sciences and Community Dental Care, Faculty of Dentistry

University of Hong Kong

Hong Kong 999077, China

Prof. Dr. Z.C. Song

Department of Periodontology

Ninth People's Hospital, Shanghai Jiao Tong University School of Medicine
Shanghai 200125, China

Prof. Dr. Y.C. Li, Prof. Dr. C.E. Wen
School of Engineering
RMIT University
Melbourne Victoria 3001, Australia
E-mail: cuie.wen@rmit.edu.ac (C.E. Wen)

Prof. Dr. Y.S. Zhou
Department of Prosthodontics
Peking University School and Hospital of Stomatology, National Center for Stomatology, National Engineering Research Center of Oral Biomaterials and Digital Medical Devices, National Clinical Research Center for Oral Disease, Beijing Key Laboratory of Digital Stomatology, Research Center of Engineering and Technology for Computerized Dentistry Ministry of Health
Beijing 100081, China
E-mail: kqzhouysh@hsc.pku.edu.cn.

**Supplementary Figures**

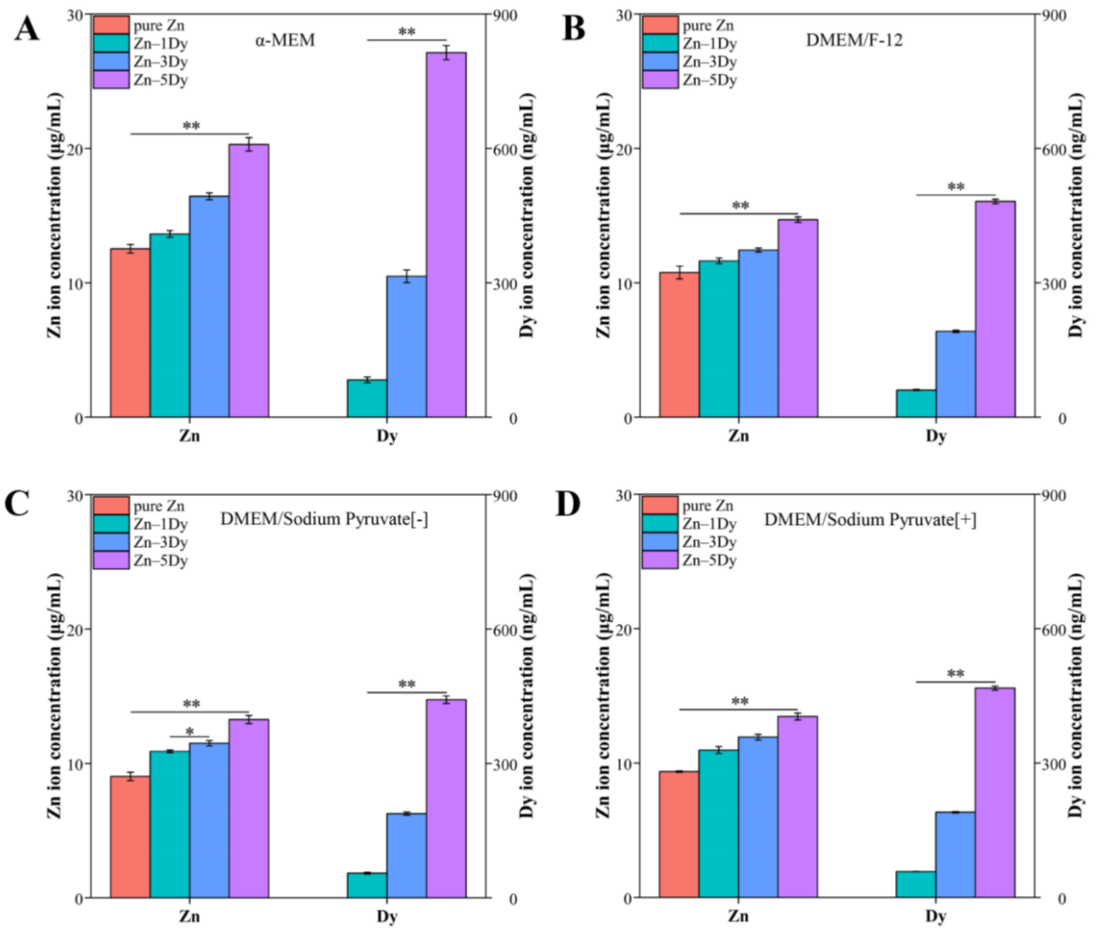


**Figure S1(A–D)** Concentrations of $Zn^{2+}$ and $Dy^{2+}$ ions in Zn-xDy (x = 0, 1, 3, and 5) extracts from different media: **(A)** α-MEM; **(B)** DMEM/F-12; **(C)** DMEM/sodium pyruvate[−]; and **(D)** DMEM/sodium pyruvate[+]. *$p$<0.05, **$p$<0.01, ***$p$<0.001, ****$p$<0.0001.

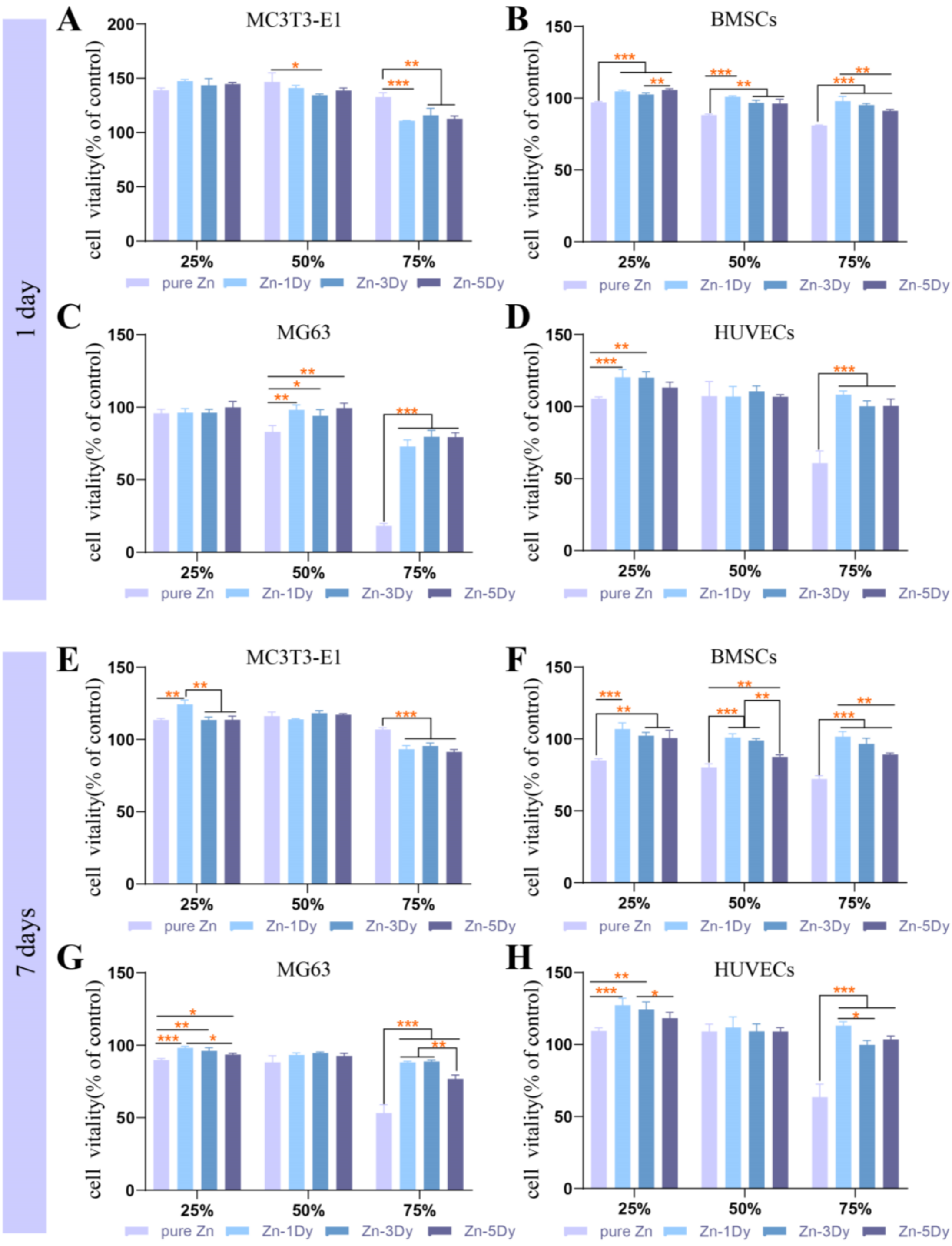


**Figure S2** Cell viability of Zn-xDy (x = 0, 1, 3, and 5) alloy extracts at 25%, 50%, and 75% concentrations after culture with four cell lines for 1 and 7 d: **(A, E)** MC3T3-E1; **(B, F)** BMSCs; **(C, G)** MG-63; and **(D, H)** HUVECs. **p*<0.05, ***p*<0.01, ****p*<0.001, *****p*<0.0001.

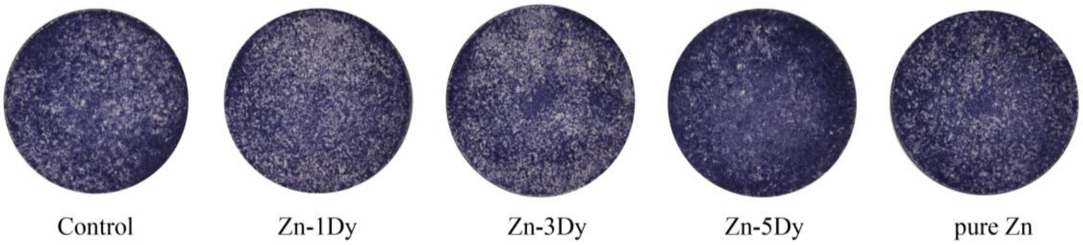


**Figure S3** Osteogenic differentiation of 25% concentration extracts of HR Zn-xDy, pure Zn, and control towards MC3T3-E1 cells.

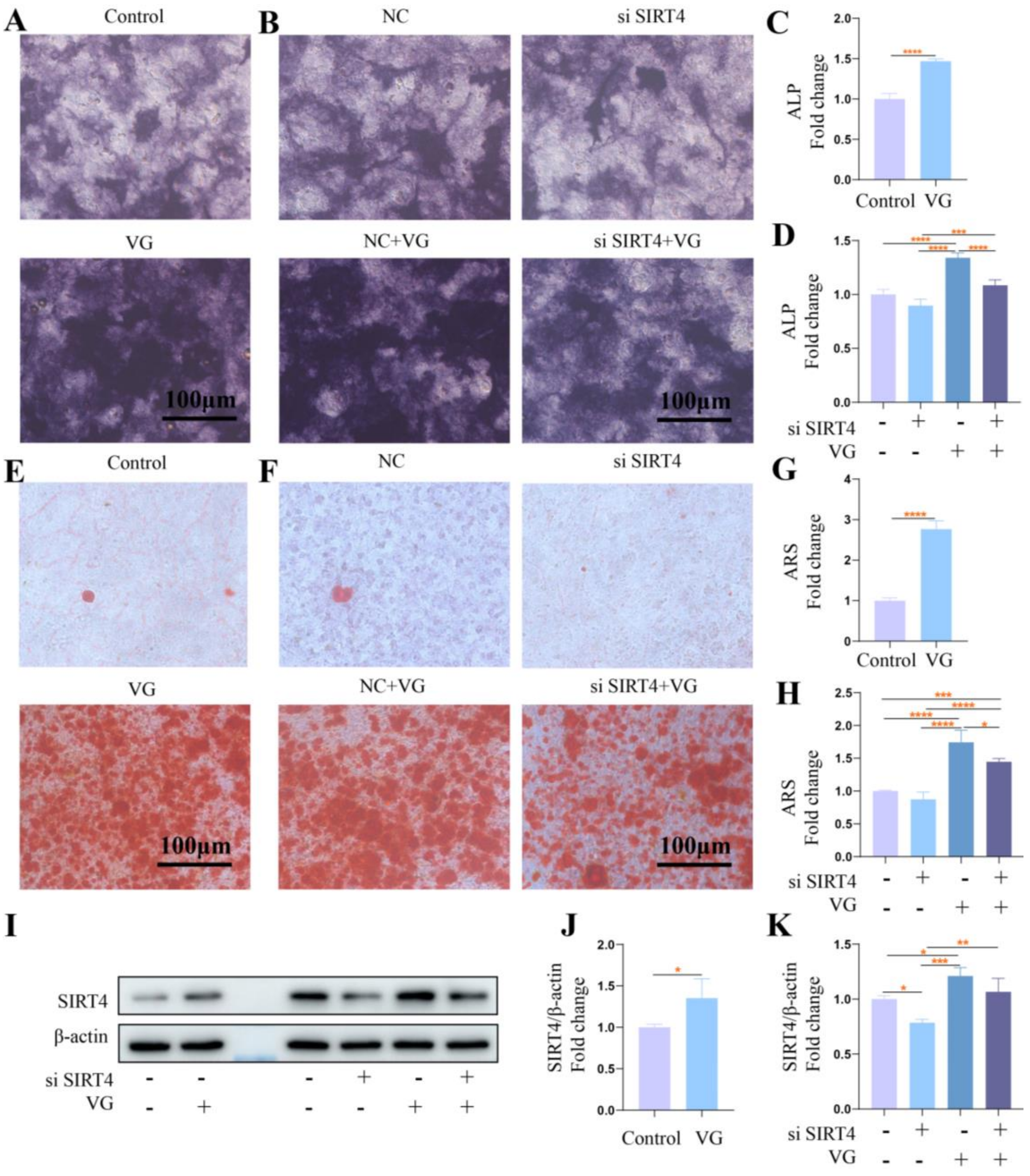


**Figure S4** SIRT4 expression increased after treating MC3T3-E1 cells with osteogenic

induction medium: **(A, B)** ALP staining; **(C, D)** quantitative analysis of ALP; **(E, F)** ARS staining; **(G, H)** quantitative analysis of ARS; **(I)** western blotting; **(J, K)** quantitative analysis of western blotting. *$p$<0.05, **$p$<0.01, ***$p$<0.001, ****$p$<0.0001.

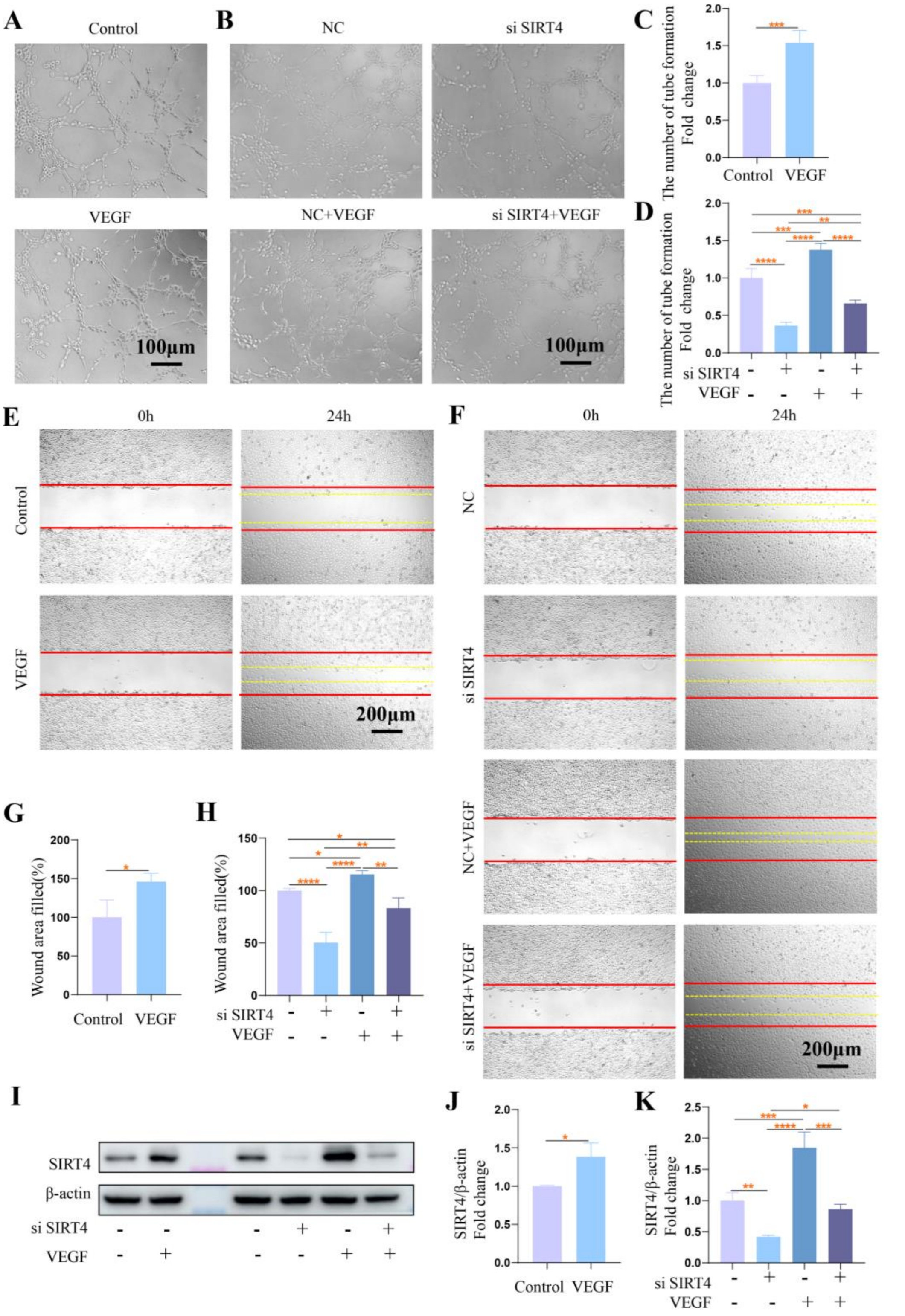

**Figure S5** SIRT4 expression increased after HUVECs were treated with VEGF: **(A, B)** tube-formation capacity; **(C, D)** quantitative tube formation; **(E, F)** cell-migration images; **(G, H)** quantitative wound-healing areas; **(I)** western blotting; **(J, K)** quantitative analysis of western blotting. *$p<0.05$, **$p<0.01$, ***$p<0.001$, ****$p<0.0001$.

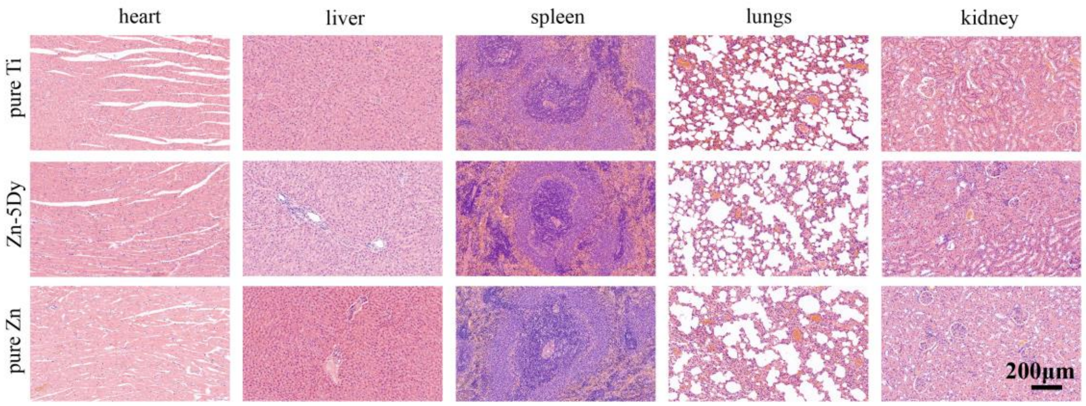


**Figure S6** Typical histological morphologies of important organic tissues in H&E sections of pure Ti, Zn-5Dy, and pure Zn 12 weeks after femur implantation.
